# Aromatase Inhibitors and Plasma Lipid Changes in Postmenopausal Women with Breast Cancer: A Systematic Review and Meta-Analysis

**DOI:** 10.3390/jcm13061818

**Published:** 2024-03-21

**Authors:** Bálint Bérczi, Nelli Farkas, Péter Hegyi, Barbara Tóth, Dezső Csupor, Balázs Németh, Anita Lukács, László Márk Czumbel, Beáta Kerémi, István Kiss, Andrea Szabó, Gábor Varga, Gábor Gerber, Zoltán Gyöngyi

**Affiliations:** 1 Department of Public Health Medicine, Medical School, University of Pécs, Szigeti út 12, 7624 Pécs, Hungary; berczi.balint84@gmail.com (B.B.); balazs.nemeth@aok.pte.hu (B.N.); istvan.kiss@aok.pte.hu (I.K.); 2 Institute for Translational Medicine, Medical School, University of Pécs, Szigeti út 12, 7624 Pécs, Hungary; nelli.farkas@aok.pte.hu (N.F.); hegyi.peter@pte.hu (P.H.); csupor.dezso@pharmacognosy.hu (D.C.); 3 Institute of Bioanalysis, Medical School, University of Pécs, Szigeti út 12, 7624 Pécs, Hungary; 4 First Department of Medicine, University of Szeged, Korányi Fasor 8-10, 6720 Szeged, Hungary; 5 Department of Pharmacognosy, University of Szeged, Eötvös u. 6, 6720 Szeged, Hungary; toth.barbara@pharmacognosy.hu; 6 Interdisciplinary Centre of Natural Products, University of Szeged, Dugonics tér 13, 6720 Szeged, Hungary; 7 Department of Public Health, Faculty of Medicine, University of Szeged, Dóm tér 10, 6720 Szeged, Hungary; lukacs.anita@med.u-szeged.hu (A.L.); szabo.andrea@med.u-szeged.hu (A.S.); 8 Department of Oral Biology, Faculty of Dentistry, Semmelweis University, Nagyvárad tér 4, 1089 Budapest, Hungary; czumbel.laszlo@semmelweis.hu (L.M.C.); keremi.beata@dent.semmelweis-univ.hu (B.K.); varga.gabor@dent.semmelweis-univ.hu (G.V.); 9 Department of Anatomy, Histology and Embryology, Faculty of Medicine, Semmelweis University, Tűzoltó u. 58, 1094 Budapest, Hungary; gerber.gabor@med.semmelweis-univ.hu

**Keywords:** breast cancer, lipids, anastrozole, exemestane, letrozole, meta-analysis

## Abstract

**Background:** Women are typically diagnosed with estrogen receptor-positive breast cancer around the postmenopausal period when declining estrogen levels initiate changes in lipid profiles. Aromatase inhibitors (AI) are used to prevent the progression of cancer; however, a further reduction in estrogen levels may have detrimental effects on lipid levels, which was our working hypothesis. **Methods:** Our meta-analysis was conducted on the lipid profiles of postmenopausal breast cancer patients at baseline and at different treatment time points. **Results:** We identified 15 studies, including 1708 patients. Studies using anastrozole (ANA), exemestane (EXE), letrozole (LET), and tamoxifen (TMX) were involved. Subgroup analyses revealed that 3- and 12-month administrations of LET and EXE lead to negative changes in lipid profiles that tend to alter the lipid profile undesirably, unlike ANA and TMX. **Conclusions:** Our results suggest that, despite statistically significant results, EXE and LET may not be sufficient to cause severe dyslipidemia in patients without cardiovascular comorbidities according to the AHA/ACC Guideline on the Management of Blood Cholesterol. However, the results may raise the question of monitoring the effects of AIs in patients, especially those with pre-existing cardiovascular risk factors such as dyslipidemia.

## 1. Introduction

Breast cancer remains a pivotal health challenge globally. Approximately 60–70% of breast cancer is estrogen receptor-positive (ER+) and is usually diagnosed around the peri- or postmenopausal period [1,2,3,4]. This is a topical issue due to the decline in the mean age of menopause in low-income and middle-income countries [5]. ER+ breast cancer often necessitates endocrine therapy as a cornerstone of treatment. Aromatase inhibitors (AIs) have emerged as a vital component of this therapeutic approach, offering a survival advantage by inhibiting the enzyme aromatase, thereby reducing estrogen levels and inhibiting the growth of breast cancer cells [6].

However, the impact of AIs on metabolic parameters, especially plasma lipid profiles, has garnered attention due to the potential implications for cardiovascular health in this vulnerable population. The relationship between estrogen, lipid metabolism, and cardiovascular health is intricate, with estrogen believed to play a protective role in cardiovascular function. Given that AIs significantly reduce estrogen levels, understanding the consequent effects on plasma lipids is crucial, as alterations in lipid profiles could predispose individuals to cardiovascular diseases, which are of particular concern in postmenopausal women with breast cancer, who may already be at an increased risk due to age, cancer therapy, and possible pre-existing conditions.

Estrogen is essential for hepatic ApoB100/ApoE receptor production, which eliminates low-density lipoprotein cholesterol (LDL-C) from the liver and is responsible for the conversion of cholesterol into cholic acid by increasing hepatic 7α-hydroxylase activity and decreasing hepatic lipase activity to maintain the high-density lipoprotein cholesterol (HDL-C) sub-fraction HDL2. These findings have been supported by the elimination of dyslipidemia with estrogen replacement [7,8,9,10,11,12,13,14]. Among healthy postmenopausal women, declining estrogen due to decreased folliculogenesis is coupled with elevated serum total cholesterol (TC), LDL-C, triglycerides (TG), and decreased HDL-C [15,16,17,18]. In this population, cardiovascular diseases (CVDs) are one of the leading causes of comorbidities and death [19,20]. Therapeutic options to prevent ER+ tumor progression include receptor antagonism with selective estrogen modulators and the inhibition of the conversion of androgens into estrogen using AIs, providing estrogen reduction [21]. AIs can be divided into two categories. Type I AIs (exemestane, EXE) are steroidal compounds designed to maintain permanent inhibition by forming covalent bonds with aromatase, a condition only reversed by the newly synthesized aromatase. However, Type II AIs, anastrozole (ANA) and letrozole (LET), are non-steroidal reversible competitive inhibitors, and their constant presence is necessary for aromatase inhibition and estrogen reduction [21].

The tolerability of AIs has been analyzed, and most of the adverse effects are consistent with estrogen deficiency—compared with tamoxifen (TMX), Type I and II AIs have demonstrated an increased risk of inflammatory rash, arthralgia, and diarrhea. Regarding Ais, to date, three meta-analyses have found a significant positive association between cardiovascular adverse events and Ais compared with TMX [22,23,24]. Despite the statistically significant results, lipid levels have not entirely been statistically investigated for EXE, ANA, and LET in a comparative study.

This systematic review and meta-analysis statistically encompasses lipid alterations reported in clinical trials of postmenopausal ER+ breast cancer patients prospectively administered EXE, ANA, and LET. The purpose of our systematic review and meta-analysis was to determine the effect of Ais on serum lipid values among patients with elevated risk of malignancy or with existing ER+ breast cancer and to contribute to a more informed approach to managing the treatment of hormone receptor-positive breast cancer to optimize overall patient health outcomes.

## 2. Materials and Methods

Our meta-analysis was reported following the Preferred Reporting Items for Systematic Reviews and Meta-Analyses (PRISMA) statement and registered in PROSPERO; the registration number is CRD42019116159 [25].

### 2.1. Literature Search Strategy

#### 2.1.1. Web of Science

To perform our search on Web of Science, we used the reference database 2023 Clarivate Analytics, Web of Science Group, Web of Science (Clarivate PLC, Saint Helier, UK). We conducted our search on 15 June 2023. Our search used medical subject headings (MeSHs) and terms. We used the function “TOPIC, (searching across the following fields within a record: Title, Abstract, Author Keywords, Keywords Plus^®^), the algorithm was: (aromatase inhibitor) AND TOPIC: (lipid) AND TOPIC: (breast cancer)”. The type of database was “All Databases” (searching across all subscribed resources). We did not use any time restriction (all years: 1975–2023). A detailed search query for Web of Science can be seen in Supplementary Table S1.

#### 2.1.2. MEDLINE

We used the reference database of the National Center for Biotechnology Information (NCBI; Bethesda, MD, USA) National Library of Medicine. We conducted our search via PubMed on 15 June 2023, where the algorithm was “((aromatase inhibitor) AND (lipid)) AND (breast cancer)” in the Advanced Search Builder. We used no field (“All Fields”) or time restrictions. A detailed search query for MEDLINE can be seen in Supplementary Table S2.

#### 2.1.3. Embase

The reference database was the 2023 Elsevier Life Sciences Excerpta Medica Database (Embase; Amsterdam, The Netherlands) via Wiley. Here, the date of the search was also 15 June 2023. In the advanced search interface, we used the following algorithm: aromatase AND inhibitor AND lipid AND breast AND cancer without any field (“All Fields”) or time (“Limit to 1966–2023”) restrictions. A detailed search query for Embase can be seen in Supplementary Table S3.

#### 2.1.4. Cochrane Library

To conduct this search, we used the 2000–2023 database by John Wiley & Sons, Inc. (New York, NJ, USA), the Cochrane Collaboration, on 15 June 2023. In the advanced interface, we applied another algorithm without time restriction (“All Dates”): aromatase inhibitor in Title Abstract Keyword AND lipid in Title Abstract Keyword AND breast cancer in Title Abstract Keyword (Word variations have been searched). Detailed search queries for Cochrane Library can be seen in Supplementary Table S4.

All databases were accessible through the Hungarian Electronic Information Service National Programme and the University Library of Pécs. All research hits were exported in “txt”, “html”, “csv”, “xml”, “docx”, “pdf”, “xlsx”, and “bib” to record our results and in file formats the “ciw”, “nbib”, and “ris” to import and perform the study selection process by using EndNote version X7.0.2 (Thomson Reuters, Toronto, Canada). In our three-step process, we first automatically detected and eliminated duplicates. Then, we manually screened the remaining records to find additional duplicates. Matching titles, authors, publishing dates, and/or DOI numbers were used as exclusion criteria. Finally, to confirm the accuracy of identification, the remaining publications were automatically screened again to ensure no further duplicates were found.

### 2.2. Selection Criteria and Data Extraction

In this phase, we individually reviewed the abstracts and titles of records to identify full-text articles. At this point, we assessed the eligibility in five consecutive steps: screening publications by the inclusion and exclusion criteria, study selection by therapy duration, inspection of data usability, and selection based on the dimensions of lipid values. Eligibility criteria were based on patients, interventions, comparisons, outcomes, and study design (PICOS), and for inclusion, full-text publications had to meet our PICO criteria [26].

Patients were postmenopausal women with existing breast cancer (primary, early, or advanced), and patients with an increased risk of breast cancer (history of benign breast cancer, prior malignant breast cancer, prior ductal carcinoma in situ (DCIS), or prior lobular carcinoma in situ (LCIS) or a history of lobular neoplasia or atypical ductal hyperplasia). The intervention was the administration of aromatase inhibitors in separate study populations for ANA, LET, and/or EXE. Comparisons were the same patients before and after the administration of aromatase inhibitors. The outcomes were serum TC, LDL-C, HDL-C, and TG levels at baseline; different treatment time points of intervention; and the mean difference (%) between the baseline and treatment time points within each treatment.

### 2.3. Risk of Bias Assessment and Data Presentation

The procedure was performed for all included studies following the PRISMA statement and the Cochrane Handbook for Systematic Reviews of Interventions [25,27]. Risk of bias assessment involved adequacy of randomization, concealment of allocation, blinding of patients, healthcare providers, data collectors, outcome assessors, extent of loss to follow-up, and stopping the trial early for benefit. During the selection procedure, interrater reliability statistics were performed [28]. BB and NF selected articles for inclusion. BB and GG assessed the risk of bias. ZG resolved any disputes as a third person. We extracted the first author’s name, trial name, year of publication, type of treatment arm, number of patients, age in years, and BMI. Type of malignancy, therapy duration, and serum lipid values were also obtained. Extracted data are presented in Table 1. Only English publications were included. The datasets generated during the current study are available from the corresponding authors upon request.

### 2.4. Statistical Analysis

Pre- to post-treatment differences in serum lipid values between baseline and treatment time points were evaluated. The differences in means with 95% confidence intervals (CIs) were calculated for all included studies. The random effect model by DerSimonian and Laird was used, and Cochrane’s Q and I^2^ statistics were applied to test heterogeneity, where *p* < 0.1; in all other cases, *p* < 0.05 was considered statistically significant [29].

Although we knew that a correlation existed between the datasets (before and after values), independent *t*-tests were used due to a lack of information about the correlation coefficients. With this procedure, we did not increase the possibility of type I errors because t-values were underestimated and calculated from correlated datasets. The following equations were used.

Equation (1) demonstrates the first step, the calculation of the s^2^ value:

(1) $$s^{2}=\frac{{(n}_{1}-1)*{{sd}_{1}}^{2}+(n_{2}-1)*{{sd}_{2}}^{2}}{n_{1}+n_{2}-2}$$

Equation (2) represents the second step, the calculation of the t-value:

(2) $$\frac{{mean}_{1}-{mean}_{2}}{\sqrt{\frac{s^{2}}{n_{2}}+\frac{s^{2}}{n_{2}}}}$$

In Equations (1) and (2), n_1_ and n_2_ are the number of patients in each group. SDs are represented by sd^1^ and sd^2^. Variance is s^2^. In Equation (2), mean_1_ and mean_2_ demonstrate the average values of each sample set [29].

**Table 1:** Detailed characteristics of included studies. ANA, anastrozole; DCIS, ductal carcinoma in situ; ER+, estrogen receptor-positive; EXE, exemestane; HDL-C, high-density lipoprotein cholesterol; HR+, hormone receptor-positive; LDL-C, low-density lipoprotein cholesterol; LET, letrozole; NR, not reported; SD, standard deviation; TC, total cholesterol; TG, triglycerides; TMX, tamoxifen.

Source (Trial Name)	Year of Publication	Location	Experimental Arm Therapy (Daily Dose)	Number of Patients Included	Age in Years (by the Experimental Arm)	BMI	Type of Disease	Overall Therapy Duration (Months)	Outcome (mg/dL)
Lønning PE et al. [30]	2005	Norway	EXE (25 mg)	EXE: 64	mean [range] EXE: 60 [46–73]	no. of patients: underweight: 3; normal: 28; overweight: 28; obese: 13	ER+ breast cancer or DCIS	24	TC, LDL-C, HDL-C, TG
Francini G et al. [31]	2006	Italy	EXE (25 mg) TMX (20 mg)	EXE: 28 TMX: 27	mean [SD] 61.89 [4.45]	mean [SD] 29.17 [2.12]	ER+ resected breast cancer	12	TC, LDL-C, HDL-C, TG
Montagnani A et al. [32]	2008	Italy	EXE (25 mg) TMX (20 mg)	EXE: 33 TMX: 35	mean [SD] 61.6 [7.2]	mean [SD] 28.1 [1.3]	ER+ resected breast cancer	24	TC, LDL-C, HDL-C, TG
Markopoulos C et al. (TEAM Greek substudy) [33]	2008	Greece	EXE (25 mg) TMX (20 mg)	EXE: 77 TMX: 65	NR	NR	ER+ resected primary breast adenocarcinoma	12	TC, LDL-C, HDL-C, TG
Markopoulos C et al. (ATENA lipid substudy) [34]	2009	Greece	EXE (25 mg)	211	mean [range] 62.6 [40–81]	NR	operable breast cancer	24	TC, LDL-C, HDL-C, TG
Sawada S et al. [35]	2009	Japan	ANA (1 mg) TMX (20 mg)	ANA: 22 TMX: 22	mean [SD] 59.3 [5.9]	mean [SD] 23.4 [5.4]	ER+ resected breast cancer	3	TC, LDL-C, HDL-C, TG
Zidan J et al. [36]	2010	Israel	LET (2.5 mg)	52	mean [range] 56 [45–89]	NR	ER+ metastatic breast cancer	12	TC, LDL-C, HDL-C, TG
Anan K et al. [37]	2011	Japan	ANA (1 mg)	33	mean [range] 60.0 [50–86]	mean [range] 24.0 [18.1–31.0]	ER+ resected breast cancer	24	TC, LDL-C, HDL-C, TG
Bell LN et al. [38]	2012	USA	EXE (25 mg) LET (2.5 mg)	EXE 117 LET: 129	mean [range] EXE 59 [44–85], LET: 57 [38–80]	mean [SD] EXE: 29.6 [6.3] LET: 29.0	ER+ DCIS (stage 0) or stage I–III invasive breast cancer	3	TC. LDL-C, HDL-C, TG
Iwata H et al. [39]	2013	Japan	EXE (25 mg) ANA (1 mg)	EXE:149 ANA:149	mean [SD] EXE: 63.4 [9.3] ANA: 64.0 [9.0]	mean [SD] EXE: 23.0 [3.6] ANA: 23.6 [4.5]	ER+ breast cancer	12	TC, LDL-C, HDL-C, TG
Santa-Maria CA et al. (ELPh sub-analysis) [40]	2015	USA	EXE (25 mg) LET (2.5 mg)	EXE: 132 LET: 147	median [range] 59 [35,89]	median [range] 29 [17.7, 55.9]	HR+ DCIS or stage I–III breast cancer	3	TC, LDL-C, HDL-C, TG
López AM et al. [41]	2015	USA	LET (2.5 mg)	28	mean [SD] LET: 61 [8]	mean [SD] 29.2 [6.6]	>1.66% probability of developing invasive breast cancer within 5 years or prior excised LCIS	3	TC, LDL-C, HDL-C, TG
Gatti-Mays ME et al. [42]	2016	USA	EXE (25 mg)	42	mean: 59.1 range and SD are not reported	mean: 29.3 range and SD are not reported	elevated Gail Model risk, prior lobular neoplasia, atypical ductal hyperplasia, or resected DCIS	24	TC, LDL-C, HDL-C, TG
Al-Biati HA et al. [43]	2016	Iraq	LET (2.5 mg) TMX (20 mg)	LET: 15 TMX: 15	mean [SD] 54.3 [3.47]	mean [SD] 28.1 ± 1.8	intracystic papillary breast carcinoma	3	TC, HDL-C, TG
Tian W et al. [44]	2017	China	LET (2.5 mg) ANA (1 mg) EXE (25 mg)	LET: 38 ANA: 51 EXE: 27	mean [range] EXE: 59.8 [52–77] LET: 59.5 [48–79]	mean [SD] EXE: 24.78 (3.81) LET: 23.33 (3.11)	HR+ early-stage breast cancer	24	TC, LDL-C, HDL-C, TG

## 3. Results

### 3.1. Literature Search Results

**Figure 1:**
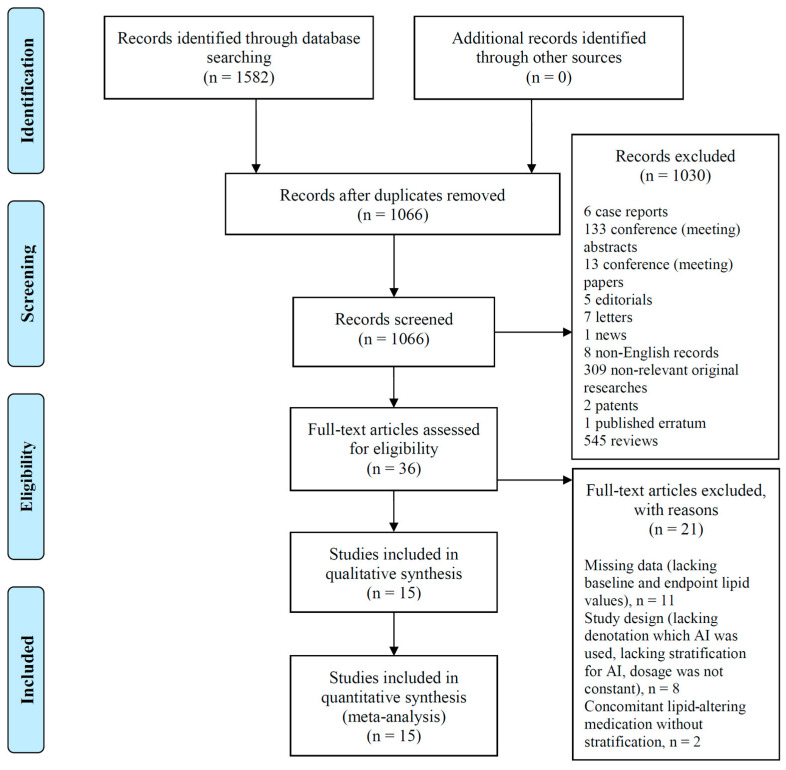
PRISMA flow diagram. PRISMA (Preferred Reporting Items for Systematic Reviews and Meta-Analyses) flow chart of study inclusion and exclusion, www.prisma-statement.org [25].

A systematic search of the Web of Science, MEDLINE, Embase, and Cochrane Library databases identified 415, 294, 771, and 76 hits, respectively. We used these 1582 records during the selection process, as shown in the PRISMA diagram in Figure 1.

Duplicates were excluded, and the remaining 1066 records were screened. Based on our PICO format, 36 studies were included for eligibility testing, and 21 studies were excluded according to our multistep selection process for the following reasons: lacking baseline and endpoint lipid values (*n* = 11) and study design (lacking denotation on which AI was used, lacking stratification for AI, or dosage was not constant; *n* = 8); studies with concomitant interventions that could impact serum lipid levels were also eliminated if the interventional group was not stratified in a distinct subgroup (*n* = 2). Finally, 15 eligible prospective studies were identified with 1708 patients enrolled within EXE, ANA, or LET experimental therapy arms. Data were collected for further subgroup analysis if an eligible study included combination therapy with selective estrogen modulator tamoxifen (TMX) in a distinct arm.

Interrater reliability statistics were performed for study inclusion when individual decisions needed to be compared [28]. Our calculation yielded a Cohen’s coefficient (κ) of 0.87 and 94.12% agreement. Similarly, the risk of bias evaluation also provided high Cohen’s coefficient (κ = 0.81) and agreement values (90.91%).

### 3.2. Characteristics of Eligible Studies

The final eligible publications were published from July 2005 to October 2017 [30,31,32,33,34,35,36,37,38,39,40,41,42,43,44]. Fasting serum lipid levels were mainly assessed in Clinical Laboratory Improvement Amendment (CLIA)-approved laboratories. The detailed characteristics of the included studies are presented in Table 1. Two reviewers, BB and GG, independently analyzed the selected studies, and ZG, as a third reviewer, resolved any disagreements. Randomization was reported in 11 out of 15 studies. Two studies mentioned randomization using computer-generated sequences, where the individuals performing the randomization and carrying out the study measurements were blinded. Three studies were double-blinded. Five were open-labeled, and five had unclear blinding. One study ended prematurely because of an early publication. Selective reporting was not detected in any of the publications. Patient drop-outs exceeded 30% in three studies. One publication reported initial endocrine and sequential therapy after TMX in its retrospective study, but the exact treatment and timing were not specified. In one trial, 72% of the patients received adjuvant and conventional treatments before letrozole. Publication bias was examined by visual inspection of funnel plots, where the standard error was plotted against the difference in means. The detailed results of risk of bias assessment are shown in Supplementary Table S5 and are based on the Cochrane Handbook for Systematic Reviews of Interventions [27].

### 3.3. Meta-Analysis of Aromatase Inhibitors ANA, EXE, and LET on Serum TC, LDL-C, HDL-C, and TG in 3- and 12-Month Administration Intervals

Forest plots were used to represent one type of lipid and time interval for all AIs (and for TMX, if available). Thus, eight forest plots and 32 subgroups were generated.

Three forest plots considering TC and TG are displayed in Figure 2, Figure 3 and Figure 4. The remaining five forest plots for LDL-C and HDL-C can be seen in Supplementary Figures S3, S6, S9, S12 and S15. Funnel plots were only provided for subgroups if the number of included studies was higher than two. These 15 plots are published as Supplementary Figures S1, S2, S4, S5, S7, S8, S10, S11, S13, S14 and S16–S20.

To determine and compare lipid risk categories considering fasting serum lipid TC, LDL-C, HDL-C, and TG levels at the baseline; and the 3- and 12-month treatment time points, we used the 2018 AHA/ACC Guideline on the Management of Blood Cholesterol, determining risk categories as “low”, “ideal”, “desirable”, “above desirable”, “borderline high”, “high”, and “very high” [45]. Detailed results of each analysis can be seen in Table 2.

#### 3.3.1. TC after 3-Month Administration of AIs and TMX

**Figure 2:**
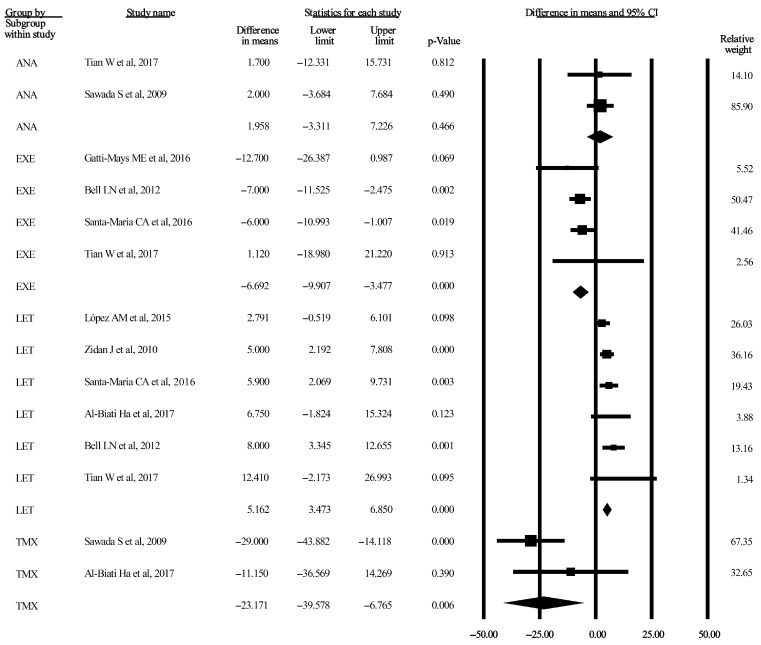
Forest plot of TC after 3-month administration of AIs and TMX. Pooled mean differences were calculated between each study’s baseline and endpoint treatment times. Studies here are stratified by AIs (ANA, EXE, and LET) and TMX measured by mg/dL (*p* < 0.05). The size of each box represents the weight contribution of the studies. The vertical lines represent the summary points for the random effect model; diamonds represent overall differences in the means of TC for each stratum. AI, aromatase inhibitor; ANA, anastrozole [35,44]; CI, confidence interval; EXE, exemestane [38,40,42,44]; LET, letrozole [36,38,40,41,43,44]; TC, total cholesterol; TMX, tamoxifen [35,43].

According to the random effect model, the pooled differences in the means between the baseline and endpoint treatment times in the ANA, EXE, and LET subgroups were 1.95 mg/dL (95% CI: −3.31–7.22 mg/dL, *p* = 0.46), −6.69 mg/dL (95% CI: −9.90–3.47 mg/dL, *p* < 0.001), and 5.16 mg/dL (95% CI: 3.47–6.85 mg/dL, *p* < 0.001), respectively [35,36,38,40,41,42,43,44]. Two publications were included in the TMX subgroup, where the pooled difference in the means was −23.17 mg/dL (95% CI: −39.57–−6.76 mg/dL, *p* = 0.006) [35,43]. The related forest plot is shown in Figure 2. The heterogeneity was unimportant for all AIs, as indicated by I^2^ values of 0% for ANA, EXE, and LET and 29.11% for TMX. The detailed results are displayed in Table 2, and the related funnel plots are shown in Supplementary Figures S1 and S2.

#### 3.3.2. TC after 12-Month Administration of AIs and TMX

One publication for this lipid type and time interval was found for the ANA subgroup, where the mean difference was 2.17 mg/dL (95% CI: −12.15–16.492 mg/dL, *p* = 0.76) [44]. The pooled differences in the means for the EXE and LET subgroups were 5.23 mg/dL (95% CI: −0.47–10.95 mg/dL, *p* = 0.07) and −0.193 mg/dL (95% CI: −9.50–9.11 mg/dL, *p* = 0.96), respectively [31,32,33,34,37,42,44]. A 12-month TMX administration was found in three publications, where the pooled difference in the means was −12.36 mg/dL (95% CI: −34.71–9.98 mg/dL, *p* = 0.27) [31,32,33]. The heterogeneity was moderate for all AIs, as indicated by I^2^ values of 48.12% and 47.43% for EXE and LET, respectively. Considerable heterogeneity was found in the TMX subgroup (I^2^ = 89.81%, *p* < 0.001). The detailed data are shown in Table 2, the forest plot for this subgroup analysis is displayed in Supplementary Figure S3, and the funnel plots are shown in Supplementary Figures S4 and S5.

#### 3.3.3. LDL-C after 3-Month Administration of AIs and TMX

Pooled differences in the means were calculated for AIs. The values for the ANA, EXE, and LET subgroups were −2.36 mg/dL (95% CI: −9.13–4.40 mg/dL, *p* = 0.49), 2.20 mg/dL (95% CI: −0.77–5.18 mg/dL, *p* = 0.14), and 4.43 mg/dL (95% CI: 2.08–6.79 mg/dL, *p* < 0.001), respectively [35,36,38,40,42,44]. One study was included as a TMX subgroup, and the difference in the means was −31.00 mg/dL (95% CI: −46.90–−15.09 mg/dL, *p* < 0.001) [34,40]. The related forest plot is shown in Supplementary Figure S6. Heterogeneity was unimportant in the ANA, EXE, and TMX subgroups, as indicated by I^2^ values of 0%. However, heterogeneity was moderate for the LET subgroup (I^2^ = 61.14%). Detailed data are presented in Table 2. Funnel plots are shown in Supplementary Figures S7 and S8.

#### 3.3.4. LDL-C after 12-Month Administration of AIs and TMX

One publication for this lipid type and time interval was found for the ANA subgroup, where the mean difference was −3.48 mg/dL (95% CI: −14.72–7.76 mg/dL, *p* = 0.54) [44]. The pooled differences in the means for the EXE and LET subgroups were 11.66 mg/dL (95% CI: 1.85–21.48 mg/dL, *p* = 0.02) and 1.13 mg/dL (95% CI: −0.80–3.07 mg/dL, *p* = 0.25), respectively [31,32,33,34,36,42,44]. A 12-month TMX administration was found in three publications, where the pooled difference in the means was −7.44 mg/dL (95% CI: −21.26–6.37 mg/dL, *p* = 0.29) [31,32,33]. The heterogeneity was insignificant (I^2^ = 0%) for ANA and LET and substantial for EXE and TMX, indicated by I^2^ values of 73.13% and 75.64%, respectively. The detailed data are shown in Table 2, the forest plot for this subgroup analysis is displayed in Supplementary Figure S9, and the funnel plots are shown in Supplementary Figures S10 and S11.

#### 3.3.5. HDL-C after 3-Month Administration of AIs and TMX

Pooled differences in the means were calculated for AIs. The values for the ANA, EXE, and LET subgroups were 5.39 mg/dL (95% CI: 2.71–8.07 mg/dL, *p* < 0.001), −7.05 mg/dL (95% CI: −9.72–4.38 mg/dL, *p* < 0.001), and 0.53 mg/dL (95% CI: −2.29–3.36 mg/dL, *p* = 0.71), respectively [35,36,38,40,41,42,43,44]. Two studies were included in the TMX subgroup, and the mean difference was 2.61 mg/dL (95% CI: −0.19–5.41 mg/dL, *p* = 0.06) [35,36,37,38,39,40,41,42,43]. The related forest plot is shown in Supplementary Figure S12. Heterogeneity was unimportant in the ANA, EXE, and TMX subgroups, as indicated by I^2^ values of 0%. However, heterogeneity was considerable for the LET subgroup (I^2^ = 94.45%). Detailed data are presented in Table 2. Funnel plots are shown in Supplementary Figures S1 and S13.

#### 3.3.6. HDL-C after 12-Month Administration of AIs and TMX

One study was included for the ANA subgroup, and the mean difference between the baseline and treatment endpoint times was 2.32 mg/dL (95% CI: −3.20–7.84 mg/dL, *p* = 0.41) [44]. The random effect model was used to calculate the pooled differences in the means for the EXE, LET, and TMX subgroups, and the values were −7.44 mg/dL (95% CI: −11.68–−3.19 mg/dL, *p* = 0.001), 2.03 mg/dL (95% CI: 1.28–2.79 mg/dL, *p* < 0.001), and −0.76 mg/dL (95% CI: −3.85–2.33 mg/dL, *p* = 0.62), respectively [31,32,33,34,36,42,44]. The related forest plot is shown in Supplementary Figure S15. Heterogeneity was insignificant for ANA, LET, and TMX, as indicated by I^2^ values of 0%. The heterogeneity for the EXE subgroup was considerable (I^2^ = 82.12%). Detailed data are displayed in Table 2, and funnel plots are shown in Supplementary Figure S16.

#### 3.3.7. TG after 3-Month Administration of AIs and TMX

**Figure 3:**
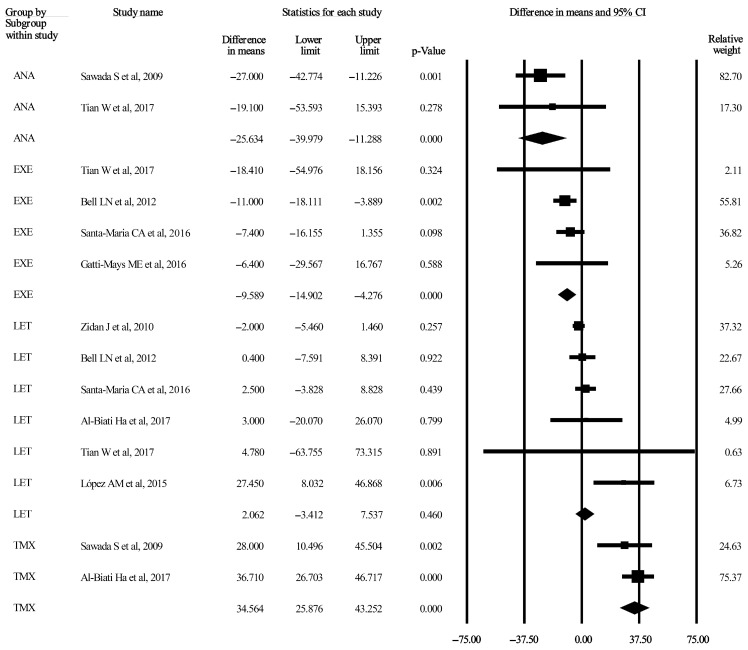
Forest plot of TG after 3-month administration of AIs and TMX. Pooled mean differences were calculated between each study’s baseline and endpoint treatment times. Studies here are stratified by AIs (ANA, EXE, and LET) and TMX measured by mg/dL (*p* < 0.05). The size of each box represents the weight contribution of the studies. The vertical lines represent the summary points for the random effect model; diamonds represent overall differences in the means of TG for each stratum. AI, aromatase inhibitor; ANA, anastrozole [35,44]; CI, confidence interval; EXE, exemestane [38,40,42,44]; LET, letrozole [36,38,40,41,43,44]; TG, triglyceride; TMX, tamoxifen [35,43].

The pooled differences in the means were calculated for AIs using the random effect model. The values for the ANA, EXE, and LET subgroups were −25.63 mg/dL (95% CI: −39.97–−11.22 mg/dL, *p* < 0.001), −9.58 mg/dL (95% CI: −14.90–−4.27 mg/dL, *p* < 0.001), and 2.06 mg/dL (95% CI: −3.41–7.53 mg/dL, *p* = 0.46), respectively [35,36,38,40,41,42,43,44]. Two studies were included as a TMX subgroup, and the pooled difference in the means was 34.56 mg/dL (95% CI: 25.87–43.25 mg/dL, *p* < 0.001) [35,43]. The related forest plot is shown in Figure 3. Heterogeneity was unimportant in the ANA, EXE, and TMX subgroups, as indicated by I^2^ values of 0%. However, heterogeneity was moderate for the LET subgroup (I^2^ = 48.14%). Detailed data are presented in Table 2. Funnel plots are shown in Supplementary Figures S17 and S18.

#### 3.3.8. TG after 12-Month Administration of AIs and TMX

**Figure 4:**
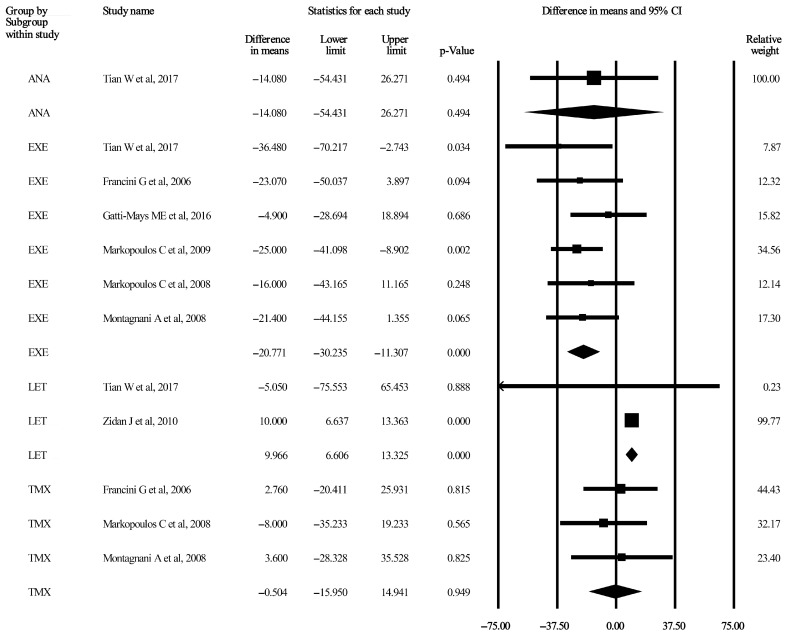
Forest plot of TG after 12-month administration of AIs and TMX. Pooled mean differences were calculated between each study’s baseline and endpoint treatment times. Studies here are stratified by AIs (ANA, EXE, and LET) and TMX measured by mg/dL (*p* < 0.05). The size of each box represents the weight contribution of the studies. The vertical lines represent the summary points for the random effect model; diamonds represent overall differences in the means of TG for each stratum. AI, aromatase inhibitor; ANA, anastrozole [44]; CI, confidence interval; EXE, exemestane [31,32,33,34,42,44]; LET, letrozole [36,44]; TG, triglycerides; TMX, tamoxifen [31,32,33].

One study was included for the ANA subgroup, and the mean difference between the baseline and treatment endpoint times was −14.08 mg/dL (95% CI: −54.43–26.27 mg/dL, *p* = 0.49) [44]. The random effect model was used to calculate the pooled differences in the means for the EXE, LET, and TMX subgroups, and the values were −20.77 mg/dL (95% CI: −30.23–−11.30 mg/dL, *p* < 0.001), 9.96 mg/dL (95% CI: 6.60–13.32 mg/dL, *p* < 0.001), and −0.504 mg/dL (95% CI: −15.95–14.94 mg/dL, *p* = 0.949), respectively [31,32,33,34,36,42,44]. The related forest plot is shown in Figure 4. Heterogeneity was not crucial for EXE, LET, and TMX, as indicated by I^2^ values of 0%. Detailed data are displayed in Table 2, and funnel plots are shown in Supplementary Figures S19 and S20.

#### 3.3.9. Subgroup Analyses of Aromatase Inhibitors LET and EXE

To see which aromatase inhibitor provided the most potent meta-analytic result, we performed additional subgroup analyses and evaluated them based on criteria.

Thirty-two subgroup analyses were conducted with AIs. Our meta-analytic criteria were the significantly different endpoint lipid levels from the baseline (*p* < 0.05), the highest number of included studies, symmetric funnel plots, a non-significant (*p* < 0.1) or unimportant (I^2^ = 0–40%) Q-value of heterogeneity, and the highest number of patients. Detailed results can be found in Table 2. Two subgroups of AIs met the aforementioned meta-analytic criteria and provided reliable interpretations.

Subgroup analysis of the LET 3-month treatment arm, which included 409 patients, demonstrated a 5.16 mg/dL (95% CI: 3.47–6.85 mg/dL, *p* < 0.001) increase in serum TC levels, where the pooled baseline value was 202.45 mg/dL (95% CI: 199.25–205.65 mg/dL) [36,38,40,41,43,44]. The subgroup analysis is shown in Figure 2. According to the guideline, the overall baseline population was classified as the “borderline high” risk category (200–230 mg/dL) for serum TC levels, which did not change at the end of the 3-month administration of LET. Detailed data are listed in Table 2.

The second AI subgroup that satisfied all the criteria and demonstrated statistically significant lipid alterations was the EXE 12-month treatment arm, which included 418 patients. Serum TG levels exhibited a 20.71 mg/dL (95% CI: −30.23–−11.30 mg/dL, *p* < 0.001) decrease, and the pooled baseline value was 129.63 mg/dL (95% CI: 117.53–141.73 mg/dL) [31,32,33,34,42,44]. The subgroup analysis is shown in Figure 4. According to the guidelines, the pooled baseline serum TG levels fell within the “desirable” risk category (<150 mg/dL), which also did not change by the end of the 12-month administration of EXE.

Of note, the observed lipid level reduction was consistent with the observed alterations of the 3-month administration of the EXE subgroup; however, the criterion regarding the maximum number of included studies was not satisfied. Here, the average of the pooled baseline serum levels of 318 patients was 116.59 mg/dL (95% CI: 104.45–128.73 mg/dL), and the difference at the endpoint was −9.58 mg/dL (95% CI: −14.90–−4.27 mg/dL, *p* < 0.001). The subgroup analysis is shown in Figure 3. The resultant directions of lipid changes in the 32 subgroup analyses are summarized in Table 3.

## 4. Discussion

Our study was intended to systematically and statistically encompass lipid changes in AI-administered populations. The meta-analysis included 15 publications and 1708 patients to reveal possible alterations in fasting lipid levels after 3- and 12-month treatment periods with ANA, EXE, and LET.

After 3- and 12-month administrations, ANA consistently increased TC and HDL-C, while LDL-C and TG decreased. Considering EXE, only LDL-C was consistently elevated, and HDL-C and TG were also reduced. Further consistency in lipid level elevation was concluded regarding LET in LDL-C, HDL-C, and TG. Interestingly, this phenomenon was noticed when analyzing the lipid levels. Except for TC, increases of similar magnitude and direction occurred over time in the subgroups if EXE was administered. By the end of the 12-month administration, the LDL-C, HDL-C, and TG levels increased by 5.3-, 1.05-, and 2.16-fold, respectively, compared with the levels during the 3-month interval; however, subgroup TG completely satisfied all of the meta-analytical criteria. A possible explanation for this phenomenon could be the irreversible binding of EXE as a false substrate to the aromatase enzyme.

In contrast, non-steroidal inhibitors ANA and LET reversibly bind to the active site of the aromatase enzyme, inhibiting the transformation of androstenedione into estrone and testosterone into estradiol [46]. Subgroup TMX decreased TC and LDL-C after 3- and 12-month administrations. This result is due to TMX having an agonistic estrogenic effect, and it is beneficial to lipid levels [47].

Considering postmenopausal dyslipidemia, further estrogen reduction may contribute to CVD risk, as bilateral ovariectomy studies have already demonstrated this association [48,49]. Treatment guidelines involve third-generation AIs. Thus, patients are typically administered them at least once, which raises concerns about the aggravation of dyslipidemia-derived CVD risks [50]. The lipid-altering effect of estrogen receptor modulators was also analyzed, and the studies concluded that tamoxifen (TMX) reduced lipoprotein(a); TC and LDL-C were decreased by raloxifene and fulvestrant after a 3-year trial [51,52,53,54].

After performing the 32 subgroup analyses and based on our meta-analytic criteria, we found that two of the subgroups provided the highest level of evidence based on heterogeneity, significance, patient number, and funnel plot symmetry. TC was significantly elevated in 409 patients by LET after the 3-month treatment, and TG was decreased in 418 patients after the 12-month administration of EXE. However, the risk category was unchanged. One or more meta-analytical criteria were not satisfied in the 30 subgroups. Furthermore, among the TMX subgroups, only the 3-month administration arm exhibited a significant decrease in TC levels; however, the number of included studies and patients and the heterogeneity did not satisfy our meta-analytical criteria.

Regarding the overall risk classification, only five subgroups exhibited alterations in the risk category; therefore, changes did not occur in 85% of the subgroups. Furthermore, there was no significant association between lipid levels and the duration of treatment. The strength of our meta-analysis was that results were further evaluated based on meta-analytic criteria, wherein EXE and LET provided the most potent evidence regarding TC and TG, respectively. In their meta-analysis, Yang et al. [55] reported that exemestane administration increased LDL-C (4.42 mg/dL) levels and decreased HDL-C (−6.03 mg/dL) and TC (−5.40 mg/dL) levels, whereas EXE lowered TG levels (−14.60 mg/dL) in a shorter-than-one-year period. These data align with our findings in the cases of LDL-C and HDL-C, but a decrease in TC was reinforced at three months, unlike the 12-month treatment in our analysis. The other difference is that we concluded that the TG level was significantly lower after the 12-month treatment with EXE.

To summarize the analyses of the 32 subgroups, lipid changes during the 3-month administration were mainly consistent with the 12-month administration of AIs and TMX, where LET and EXE changed the lipid profile rather undesirably. Desirable lipid changes can only be found in the 3- and 12-month ANA and TMX treatment arms.

This meta-analysis has limitations mainly due to the analyzed studies. Several eligible studies were statistically heterogeneous, with occasionally missing values. Consequently, several selection steps had to be established to manage these missing data to construct our meta-analysis, resulting in fewer included studies and patients. Furthermore, lipid values were primarily measured in CLIA-approved laboratories; however, there were some studies in which the methodology, standards, and measurement locations still needed to be published. Nevertheless, the mentioned weaknesses were handled accordingly, resulting in a robust and reliable analysis.

Based on the provided evidence, this study is the first comparative meta-analysis to evaluate the effects of third-generation steroidal EXE and non-steroidal ANA and LET on serum lipid TC, LDL-C, HDL-C, and TG levels. Due to the detailed analyses of the included studies, our investigation successfully established a baseline population, handling confounding factors so that the effects of AIs and TMX on serum lipid levels could be determined.

In reviewing state-of-the-art treatment protocols, the aromatase inhibitors and tamoxifen drugs did not have any precautions for use regarding possible undesirable changes in the blood lipid profile [56]. Our current meta-analysis studied a population where lipid levels were essentially normal. Conversely, we observed a significant deterioration in the lipid profile after letrozole and exemestane treatment. Still, this change in a population with initially normal blood lipid values does not require a blood-lipid-regulating treatment. Although we have no evidence, it is conceivable that patients treated with LET or EXE with an initially unfavorable lipid profile warrant further attention.

## 5. Conclusions

In conclusion, our systematic review and meta-analysis have shed light on the intricate relationship between AI therapy and lipid profiles in postmenopausal women with estrogen receptor-positive breast cancer. Through the analysis of 15 studies involving 1708 patients, we observed that specific AIs, namely, letrozole and exemestane, are associated with changes in serum total cholesterol and triglyceride levels, respectively. These findings suggest that AI therapy can indeed influence lipid metabolism, although the clinical significance of these alterations appears limited within the contexts explored in our review.

The implications of our findings for clinical practice are two-fold. First, pre-existing dyslipidemia before AI treatment raises concerns about using EXE and LET in first-line therapy for ER+ breast cancer. Second, we underscore the necessity and importance of healthcare providers maintaining a vigilant approach to monitoring lipid levels in AI-treated postmenopausal patients, particularly in those with pre-existing cardiovascular risk factors or a significant family history of cardiovascular disease. The postmenopausal condition in itself is a risk factor for undesirable lipid alterations; if we add to this pre-existing dyslipidemia caused by cardiovascular risk factors, applying EXE and LET raises concerns and may further deteriorate lipid levels according to our results, in which the population was free from dyslipidemia.

Future research should further delineate the long-term cardiovascular impacts of AI therapy, exploring both lipid-dependent and lipid-independent mechanisms. Such studies are crucial for developing comprehensive, evidence-based guidelines that balance the oncologic benefits of AI therapy with the potential metabolic and cardiovascular risks.

## Figures and Tables

**Table 2:** Detailed results of AI and TMX subgroup analyses. AIs, aromatase inhibitors; ANA, anastrozole; EXE, exemestane; HDL-C, high-density lipoprotein cholesterol; LDL-C, low-density lipoprotein cholesterol; LET, letrozole; TC, total cholesterol; TG, triglyceride; TMX, tamoxifen. Risk categories can be found in the 2018 AHA/ACC Guideline on managing blood cholesterol [45].

Lipid Type	Treatment Time	AIs and TMX	Difference in Means (mg/dL)	Lower Limit	Upper Limit	*p*-Value	Number of Studies	Number of Patients at Baseline	Risk Category at Baseline	Risk Category at Endpoint	Heterogeneity Analysis		
											Q-Value	P_heterogeneity_	I^2^ (%)
TC	3 months	ANA	1.95	−3.31	7.226	0.46	2	73	borderline high	borderline high	0.001	0.96	0
		EXE	−6.69	−9.90	−3.477	<0.001	4	318	borderline high	above desirable	1.41	0.70	0
		LET	5.16	3.473	6.85	<0.001	6	409	borderline high	borderline high	4.63	0.46	0
		TMX	−23.17	−39.57	−6.76	0.006	2	37	borderline high	borderline high	1.41	0.23	29.11
	12 months	ANA	2.17	−12.15	16.49	0.76	1	51	borderline high	borderline high	0	1	0
		EXE	5.23	−0.47	10.95	0.07	6	418	borderline high	borderline high	9.63	0.08	48.12
		LET	−0.19	−9.50	9.11	0.96	2	90	borderline high	borderline high	1.90	0.16	47.43
		TMX	−12.36	−34.71	9.98	0.27	3	127	borderline high	borderline high	19.64	<0.001	89.81
LDL-C	3 months	ANA	−2.36	−9.13	4.40	0.49	2	73	above desirable	above desirable	0.43	0.51	0
		EXE	2.20	−0.77	5.18	0.14	4	318	above desirable	above desirable	1.04	0.78	0
		LET	4.43	2.08	6.79	<0.001	5	394	above desirable	above desirable	10.29	0.03	61.14
		TMX	−31.00	−46.90	−15.09	<0.001	1	22	borderline high	above desirable	<0.001	1	0
	12 months	ANA	−3.48	−14.72	7.76	0.54	1	51	above desirable	above desirable	0	1	0
		EXE	11.66	1.85	21.48	0.02	6	418	above desirable	borderline high	18.61	0.002	73.13
		LET	1.13	−0.80	3.07	0.25	2	90	above desirable	above desirable	0.66	0.41	0
		TMX	−7.44	−21.26	6.37	0.29	3	127	borderline high	borderline high	8.21	0.01	75.64
HDL-C	3 months	ANA	5.39	2.71	8.07	<0.001	2	73	desirable	high	0.62	0.43	0
		EXE	−7.05	−9.72	−4.38	<0.001	4	318	desirable	desirable	2.11	0.54	0
		LET	0.53	−2.29	3.36	0.71	6	409	desirable	desirable	90.19	0	94.45
		TMX	2.61	−0.19	5.41	0.06	2	37	desirable	desirable	0.14	0.70	0
	12 months	ANA	2.32	−3.20	7.84	0.41	1	51	desirable	desirable	<0.001	1	0
		EXE	−7.44	−11.68	−3.19	0.001	5	391	desirable	desirable	22.37	<0.001	82.12
		LET	2.03	1.28	2.79	<0.001	2	90	desirable	desirable	0.79	0.37	0
		TMX	−0.76	−3.85	2.33	0.62	2	62	desirable	desirable	0.01	0.91	0
TG	3 months	ANA	−25.63	−39.97	−11.28	<0.001	2	73	borderline high	desirable	0.16	0.68	0
		EXE	−9.58	−14.90	−4.27	<0.001	4	318	desirable	desirable	0.68	0.87	0
		LET	2.06	−3.41	7.53	0.46	6	409	desirable	desirable	9.64	0.08	48.14
		TMX	34.564	25.87	43.25	<0.001	2	37	high	high	0.71	0.39	0
	12 months	ANA	−14.08	−54.43	26.27	0.49	1	51	borderline high	borderline high	<0.001	1	0
		EXE	−20.77	−30.23	−11.30	<0.001	6	418	desirable	desirable	2.95	0.70	0
		LET	9.96	6.60	13.32	<0.001	2	90	borderline high	borderline high	0.17	0.67	0
		TMX	−0.50	−15.95	14.941	0.94	3	127	desirable	desirable	0.43	0.80	0

**Table 3:** Results of 32 subgroup analyses of 3- and 12-month AI and TMX administration.

Aromatase Inhibitors (AIs)												Tamoxifen (TMX)			
**ANA**				**EXE**				**LET**				**3-Months**			
**3-Months**				**3-Months**				**3-Months**							
**TC**	**LDL-C**	**HDL-C**	**TG**	**TC**	**LDL-C**	**HDL-C**	**TG**	**TC**	**LDL-C**	**HDL-C**	**TG**	**TC**	**LDL-C**	**HDL-C**	**TG**
↑	↓	↑	↓	↓	↑	↓	↓	↑	↑	↑	↑	↓	↓	↑	↑
**12-Months**				**12-Months**				**12-Months**				**12-Months**			
**TC**	**LDL-C**	**HDL-C**	**TG**	**TC**	**LDL-C**	**HDL-C**	**TG**	**TC**	**LDL-C**	**HDL-C**	**TG**	**TC**	**LDL-C**	**HDL-C**	**TG**
↑	↓	↑	↓	↑	↑	↓	↓	↓	↑	↑	↑	↓	↓	↓	↓

Red color shows undesirable changes in lipid levels (↑: elevated lipid level at the endpoint of administration compared with baseline) according to the 2018 AHA/ACC Guidelines on managing blood cholesterol. Green color shows undesirable changes in lipid levels (↓: decreased lipid level at the endpoint of administration compared with baseline) according to the 2018 AHA/ACC Guidelines on managing blood cholesterol. AIs, aromatase inhibitors; ANA, anastrozole; EXE, exemestane; HDL-C, high-density lipoprotein cholesterol; LDL-C, low-density lipoprotein cholesterol; LET, letrozole; TC, total cholesterol; TG, triglyceride; TMX, tamoxifen.

## Data Availability

The data presented in this study are available upon request from the corresponding authors.

## Supplementary Materials

The following supporting information can be downloaded at https://www.mdpi.com/article/10.3390/jcm13061818/s1, Figure S1: Funnel plot for publication bias among studies reporting TC after 3-month administration of EXE. Figure S2: Funnel plot for publication bias among studies reporting TC after 3-month administration of LET. Figure S3: Forest plot of TC after 12-month administration of AIs and TMX. Figure S4: Funnel plot for publication bias among studies reporting TC after 12-month administration of EXE. Figure S5: Funnel plot for publication bias among studies reporting TC after 12-month administration of TMX. Figure S6: Forest plot of LDL-C after 3-month administration of AIs and TMX. Figure S7: Funnel plot for publication bias among studies reporting LDL-C after 3-month administration of EXE. Figure S8: Funnel plot for publication bias among studies reporting LDL-C after 3-month administration of LET. Figure S9: Forest plot of LDL-C after 12-month administration of AIs and TMX. Figure S10: Funnel plot for publication bias among studies reporting LDL-C after 12-month administration of EXE. Figure S11: Funnel plot for publication bias among studies reporting LDL-C after 12-month administration of TMX. Figure S12: Forest plot of HDL-C after 3-month administration of AIs and TMX. Figure S13: Funnel plot for publication bias among studies reporting HDL-C after 3-month administration of EXE. Figure S14: Funnel plot for publication bias among studies reporting HDL-C after 3-month administration of LET. Figure S15: Forest plot of HDL-C after 12-month administration of AIs and TMX. Figure S16: Funnel plot for publication bias among studies reporting HDL-C after 12-month administration of EXE. Figure S17: Funnel plot for publication bias among studies reporting TG after 3-month administration of EXE. Figure S18: Funnel plot for publication bias among studies reporting TG after 3-month administration of LET. Figure S19: Funnel plot for publication bias among studies reporting TG after 12-month administration of EXE. Figure S20: Funnel plot for publication bias among studies reporting TG after 12-month administration of TMX. Table S1: Search query for Web of Science. Table S2: Search query for MEDLINE (via PubMed). Table S3: Search query for Embase. Table S4: Search query for the Cochrane Library. Table S5: Risk of bias assessment.
