# Supplementary material for: Aromatase Inhibitors and Plasma Lipid Changes in Postmenopausal Women with Breast Cancer: A Systematic Review and Meta-Analysis
Source: J Clin Med. 2024 Mar 21;13(6):1818. doi: 10.3390/jcm13061818 (PMC10970985; doi:10.3390/jcm13061818)
Supplement: Supplementary file 1 [file jcm-13-01818-s001.zip › jcm-2905779-supplementary.pdf]

Supplementary Information

Aromatase inhibitors and plasma lipid changes in postmenopausal women with breast cancer: A systematic review and meta-analysis

Bálint Bérczi, Nelli Farkas, Péter Hegyi, Barbara Tóth, Dezső Csupor, Balázs Németh, Anita Lukács, László Márk, Czumbel, Beáta Kerémi, István Kiss, Andrea Szabó Gábor Varga, Gábor Gerber, Zoltán Gyöngyi

Index

1. **Supplementary Tables**

  1.1. Supplementary Table S1. Search query for Web of Science
  1.2. Supplementary Table S2. Search query for MEDLINE (via PubMed)
  1.3. Supplementary Table S3. Search query for Embase
  1.4. Supplementary Table S4. Search query for the Cochrane Library
  1.5. Supplementary Table S5. Risk of bias assessment

2. **Supplementary Figures**

  2.1. Supplementary Figure S1. Funnel plot for publication bias among studies reporting TC after 3-month administration of EXE
  2.2. Supplementary Figure S2. Funnel plot for publication bias among studies reporting TC after 3-month administration of LET
  2.3. Supplementary Figure S3. Forest plot of TC after 12-month administration of AIs and TMX
  2.4. Supplementary Figure S4. Funnel plot for publication bias among studies reporting TC after 12-month administration of EXE
  2.5. Supplementary Figure S5. Funnel plot for publication bias among studies reporting TC after 12-month administration of TMX
  2.6. Supplementary Figure S6. Forest plot of LDL-C after 3-month administration of AIs and TMX
  2.7. Supplementary Figure S7. Funnel plot for publication bias among studies reporting LDL-C after 3-month administration of EXE.
  2.8. Supplementary Figure S8. Funnel plot for publication bias among studies reporting LDL-C after 3-month administration of LET.
  2.9. Supplementary Figure S9. Forest plot of LDL-C after 12-month administration of AIs and TMX.
  2.10. Supplementary Figure S10. Funnel plot for publication bias among studies reporting LDL-C after 12-month administration of EXE.
  2.11. Supplementary Figure S11. Funnel plot for publication bias among studies reporting LDL-C after 12-month administration of TMX.
  2.12. Supplementary Figure S12. Forest plot of HDL-C after 3-month administration of AIs and TMX
  2.13. Supplementary Figure S13. Funnel plot for publication bias among studies reporting HDL-C after 3-month administration of EXE.
  2.14. Supplementary Figure S14. Funnel plot for publication bias among studies reporting HDL-C after 3-month administration of LET.
  2.15. Supplementary Figure S15. Forest plot of HDL-C after 12-month administration of AIs and TMX.

2.16. Supplementary Figure S16. Funnel plot for publication bias among studies reporting HDL-C after 12-month administration of EXE.

2.17. Supplementary Figure S17. Funnel plot for publication bias among studies reporting TG after 3-month administration of EXE.

2.18. Supplementary Figure S18. Funnel plot for publication bias among studies reporting TG after 3-month administration of LET.

2.19. Supplementary Figure S19. Funnel plot for publication bias among studies reporting TG after 12-month administration of EXE.

2.20. Supplementary Figure S20. Funnel plot for publication bias among studies reporting TG after 12-month administration of TMX.

3. References

1. Supplementary Tables

1.1. Supplementary Table S1. Search query for Web of Science

Reference: 2021 Clarivate Analytics, Web of Science Group, Web of Science
Available from: https://clarivate.com/webofsciencegroup/solutions/web-of-science/
Research interface: https://apps.webofknowledge.com
Type of database: "All Databases" (searching across all subscribed resources)
Type of search: "TOPIC" (searching across the following fields within a record: Title, Abstract, Author Keywords, Keywords Plus ®)
Timespan: "All years (1975–2023)"
Date of search: June 15, 2023

The database was accessible via the Hungarian Electronic Information Service National Programme and the University Library of Pécs

Date of search	Search algorithm	Results
**15/06/2023**	TOPIC: (aromatase inhibitor) AND TOPIC: (lipid) AND TOPIC: (breast cancer)	415

1.2. Supplementary Table S2. Search query for MEDLINE (via PubMed)

Reference: National Center for Biotechnology Information (NCBI), Bethesda (MD): U.S. National Library of Medicine
Available from: https://www.ncbi.nlm.nih.gov/
Research interface: https://www.ncbi.nlm.nih.gov/pubmed/advanced (PubMed Advanced Search Builder)
Type of search: "All Fields"

The database was accessible via the University Library of Pécs

Date of search	Search algorithm	Results
**15/06/2023**	((aromatase inhibitor) AND (lipid)) AND (breast cancer)	294

1.3. Supplementary Table S3. Search query for Embase

Reference: 2023 Elsevier Life Sciences, Excerpta Medica dataBASE (Embase)
Available from: https://www.elsevier.com/solutions/embase-biomedical-research
Research interface: https://www.embase.com/#search
Type of search: "All Fields"
Timespan: "Limited to <1966–2023"
Date of search: June 15, 2023

The database was accessible via the University Library of Pécs

Date of search	Search algorithm	Results
**15/06/2023**	aromatase inhibitor AND lipid AND breast cancer	771

1.4. Supplementary Table S4. Search query for the Cochrane library

Reference: 2000–2023 by John Wiley & Sons, Inc., The Cochrane Collaboration,
Available from: https://www.cochranelibrary.com/
Research interface: https://www.cochranelibrary.com/advanced-search
Type of search: “Title Abstract Keyword”
Date of search: June 15, 2023
Timespan: “All dates”

The database was accessible via the University Library of Pécs

Date of search	Search algorithm	Results
**15/06/2023**	aromatase inhibitor in Title Abstract Keyword AND lipid in Title Abstract Keyword AND breast cancer in Title Abstract Keyword (word variations have been searched)	76

All research hits were exported as ‘txt,’ ‘html,’ ‘csv,’ ‘xml,’ ‘docx,’ ‘pdf,’ ‘xlsx,’ and ‘bib’ file formats to record the results and as ‘ciw,’ ‘nbib,’ and ‘ris’ file formats to import and perform the study selection process using EndNote version X7.0.2 (Thomson Reuters).

Trials	Random sequence generation (selection bias)	Allocation concealment (selection bias)	Blinding of participants and personnel (performance bias)	Blinding of outcome assessment (detection bias)	Incomplete outcome data (attrition bias)	Selective reporting (reporting bias)	Other bias
**Lønning PE et al., 2005[1]**	?	?	-	-	-	-	-
**Francini G et al., 2006[2]**	-	-	?	-	-	-	-
**Montagnani A et al., 2008[3]**	-	-	-	-	-	-	-
**Markopoulos C et al. (TEAM Greek Substudy), 2008[4]**	?	?	+	+	-	-	-
**Markopoulos C et al (ATENA lipid substudy), 2009[5]**	?	?	+	+	+	-	-
**Sawada S et al., 2005[6]**	?	?	+	+	-	-	-
**Zidan J et al., 2010[7]**	+	+	+	+	-	-	+
**Anan K et al., 2011[8]**	?	?	+	+	+	+	-
**Bell LN et al., 2012[9]**	?	?	+	+	+	-	-
**Iwata H et al., 2013[10]**	?	?	?	?	-	-	-
**Santa-Maria CA et al., 2015[11]**	?	?	+	+	+	-	-
**López AM et al., 2015[12]**	?	?	-	-	-	-	-
**Gatti-Mays ME et al., 2016[13]**	+	+	+	+	-	-	-
**Al-Biati HA et al., 2016[14]**	+	+	+	+	-	-	+
**Tian W et al., 2017[15]**	+	+	+	+	?	-	+

1.5. Supplementary Table S5. Risk of bias assessment. ?: unclear risk of bias, -: low risk of bias, +: high risk of bias [1-15].

2. Supplementary Figures

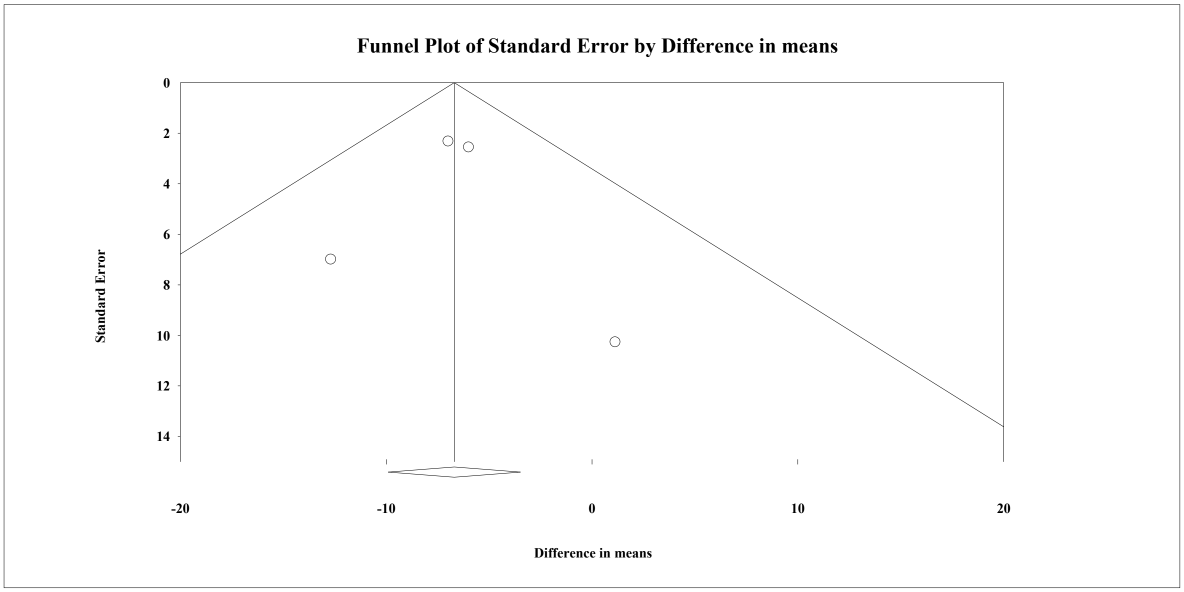

2.1. Supplementary Figure S1. Funnel plot for publication bias among studies reporting TC after 3-month administration of EXE. Visual inspection of funnel plot reveals symmetry and absence of publication bias. EXE exemestane, TC total cholesterol.

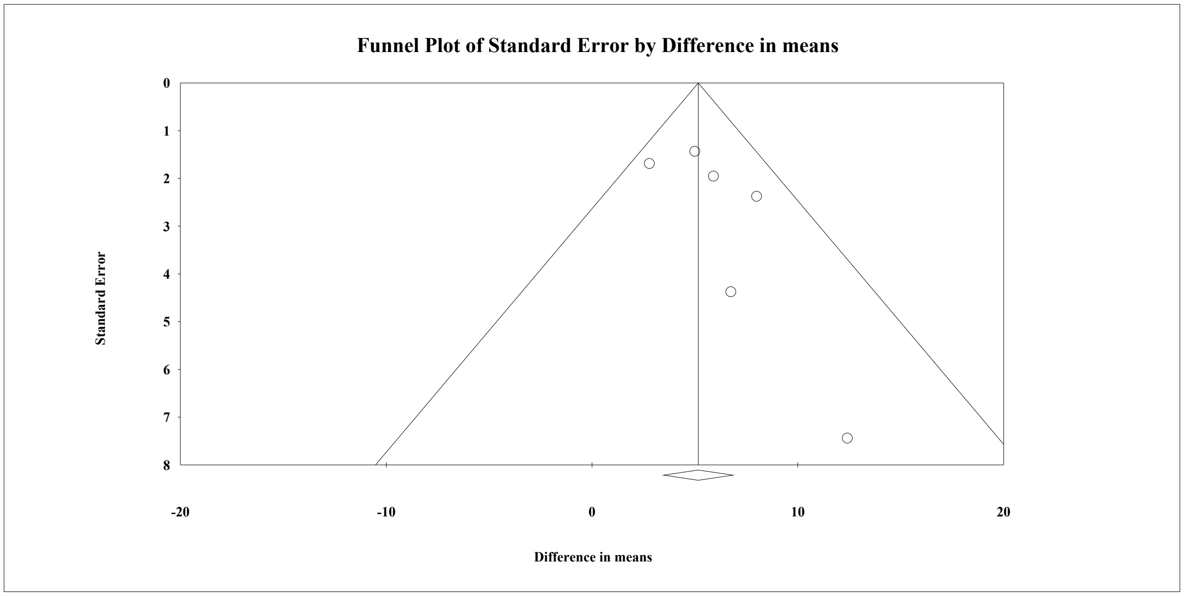

2.2. Supplementary Figure S2. Funnel plot for publication bias among studies reporting TC after 3-month administration of LET. Visual inspection of funnel plot reveals symmetry and absence of publication bias. LET letrozole, TC total cholesterol.

Group by Subgroup within study	Study name	Difference in means	Lower limit	Upper limit	p-Value	Relative weight
ANAstrozole	Tian W et al, 2017	2.170	-12.152	16.492	0.766	100.00
ANAstrozole		2.170	-12.152	16.492	0.766	
EXEmestane	Gatti-Mays ME et al, 2016	-8.600	-23.624	6.424	0.262	10.60
EXEmestane	Markopoulos C et al, 2008	-8.000	-24.216	8.216	0.334	9.46
EXEmestane	Tian W et al, 2017	-0.620	-21.350	20.110	0.953	6.38
EXEmestane	Montagnani A et al, 2008	8.300	2.764	13.836	0.003	28.86
EXEmestane	Francini G et al, 2006	8.490	2.618	14.362	0.005	27.92
EXEmestane	Markopoulos C et al, 2009	13.000	2.409	23.591	0.016	16.78
EXEmestane		5.239	-0.478	10.957	0.072	
LETrozole	Zidan J et al, 2010	-3.000	-6.120	0.120	0.060	74.13
LETrozole	Tian W et al, 2017	7.850	-7.250	22.950	0.308	25.87
LETrozole		-0.193	-9.506	9.119	0.968	
TMX, tamoxifen	Markopoulos C et al, 2008	-31.000	-43.584	-18.416	0.000	34.50
TMX, tamoxifen	Montagnani A et al, 2008	-6.300	-28.949	16.349	0.586	27.71
TMX, tamoxifen	Francini G et al, 2006	0.200	-5.469	5.869	0.945	37.79
TMX, tamoxifen		-12.364	-34.710	9.981	0.278	

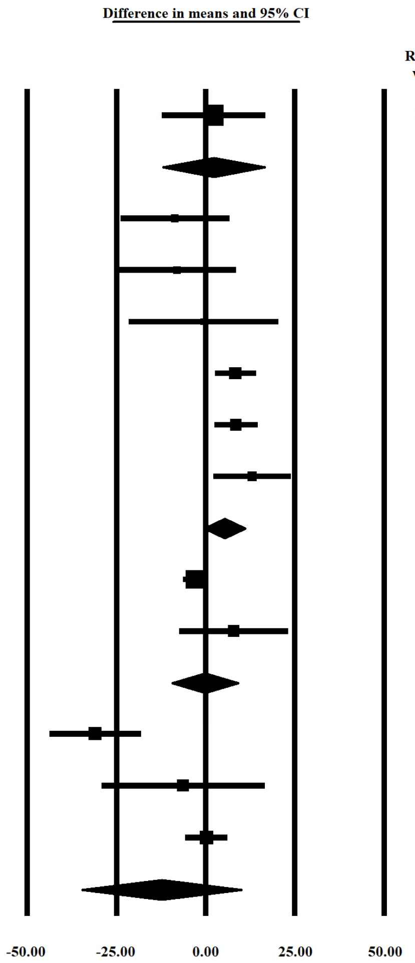

2.3. Supplementary Figure S3. Forest plot of TC after 12-month administration of AIs and TMX. Pooled differences in the means were calculated between the baseline and endpoint treatment times for each study. Studies here are stratified by AIs (ANA, EXE, LET) and TMX measured by mg/dl ($P<0.05$). Weight contribution of studies is represented by the size of each box. The vertical lines represent the points of summary for random effect model, diamonds represent overall differences in the means of TC for each strata. AI aromatase inhibitor, ANA anastrozole [44], EXE exemestane [31-34,42,44], LET letrozole [36,44], TC total cholesterol, TMX tamoxifen [31-33].

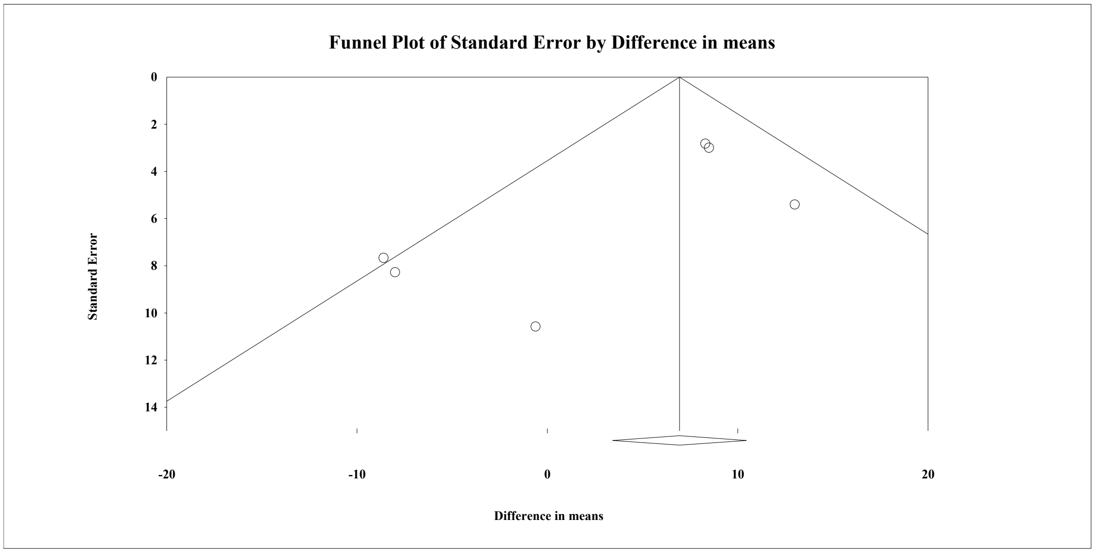

2.4. Supplementary Figure S4. Funnel plot for publication bias among studies reporting TC after 12-month administration of EXE. Visual inspection of funnel plot reveals symmetry and absence of publication bias. EXE exemestane, TC total cholesterol.

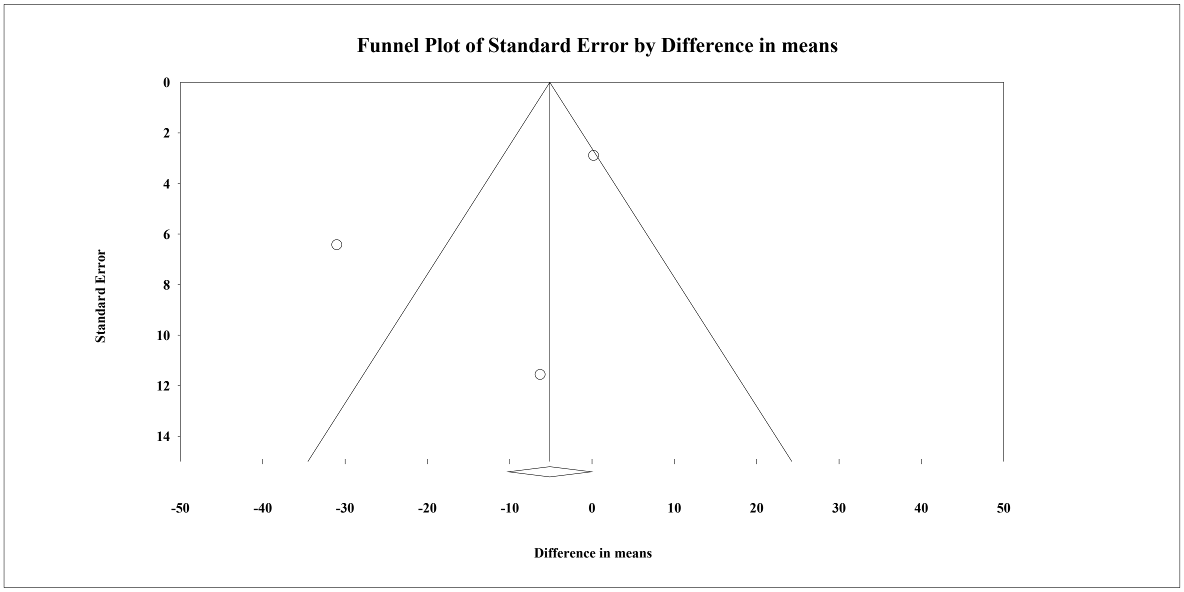

2.5. Supplementary Figure S5. Funnel plot for publication bias among studies reporting TC after 12-month administration of TMX. Visual inspection of funnel plot reveals symmetry and absence of publication bias. TC total cholesterol, TMX tamoxifen.

Group by Subgroup within study	Study name	Difference in means	Lower limit	Upper limit	p-Value	Relative weight
ANAstrozole	Sawada S et al, 2009	-4.000	-12.342	4.342	0.347	65.84
ANAstrozole	Tian W et al, 2017	0.780	-10.802	12.362	0.895	34.16
ANAstrozole		-2.367	-9.136	4.402	0.493	
EXEmestane	Gatti-Mays ME et al, 2016	-3.200	-15.967	9.567	0.623	5.45
EXEmestane	Bell LN et al, 2012	2.000	-2.274	6.274	0.359	48.60
EXEmestane	Santa-Maria CA et al, 2016	2.800	-1.751	7.351	0.228	42.88
EXEmestane	Tian W et al, 2017	6.850	-10.159	23.859	0.430	3.07
EXEmestane		2.209	-0.771	5.188	0.146	
LETrozole	López AM et al, 2015	1.536	-0.651	3.723	0.169	28.83
LETrozole	Zidan J et al, 2010	4.000	2.340	5.660	0.000	32.24
LETrozole	Santa-Maria CA et al, 2016	5.400	1.531	9.269	0.006	18.83
LETrozole	Bell LN et al, 2012	7.000	2.652	11.348	0.002	16.61
LETrozole	Tian W et al, 2017	15.080	3.087	27.073	0.014	3.50
LETrozole		4.439	2.087	6.791	0.000	
TMX, tamoxifen	Sawada S et al, 2009	-31.000	-46.908	-15.092	0.000	100.00
TMX, tamoxifen		-31.000	-46.908	-15.092	0.000	

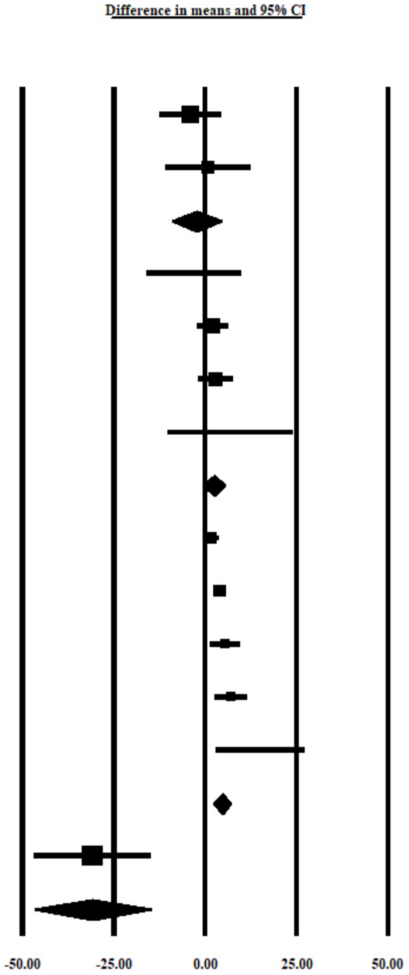

2.6. Supplementary Figure S6. Forest plot of LDL-C after 3-month administration of AIs and TMX. Pooled differences in the means were calculated between the baseline and endpoint treatment times for each study. Studies here are stratified by AIs (ANA, EXE, LET) and TMX measured by mg/dl (P<0.05). Weight contribution of studies is represented by the size of each box. The vertical lines represent the points of summary for random effect model, diamonds represent overall differences in the means of LDL-C for each strata. AI aromatase inhibitor, ANA anastrozole [35,44], CI confidence interval, EXE exemestane [38,40,42,44], LDL-C low-density lipoprotein, LET letrozole [36,38,40,41,44], TMX, tamoxifen [35].

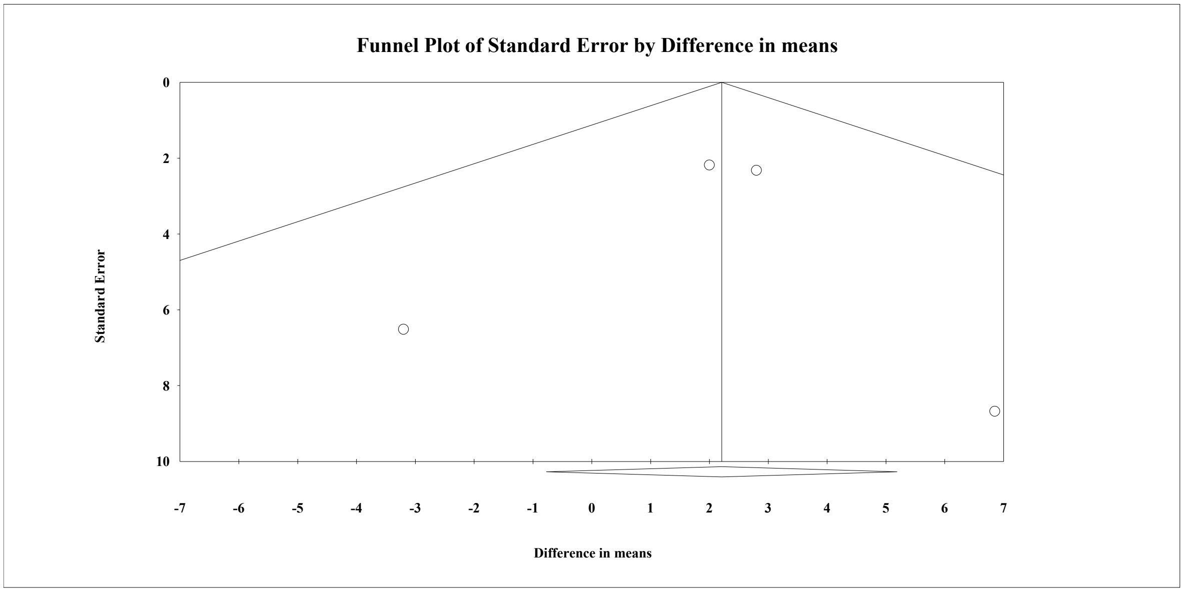

2.7. Supplementary Figure S7. Funnel plot for publication bias among studies reporting LDL-C after 3-month administration of EXE. Visual inspection of funnel plot reveals symmetry and absence of publication bias. EXE exemestane, LDL-C low-density lipoprotein cholesterol.

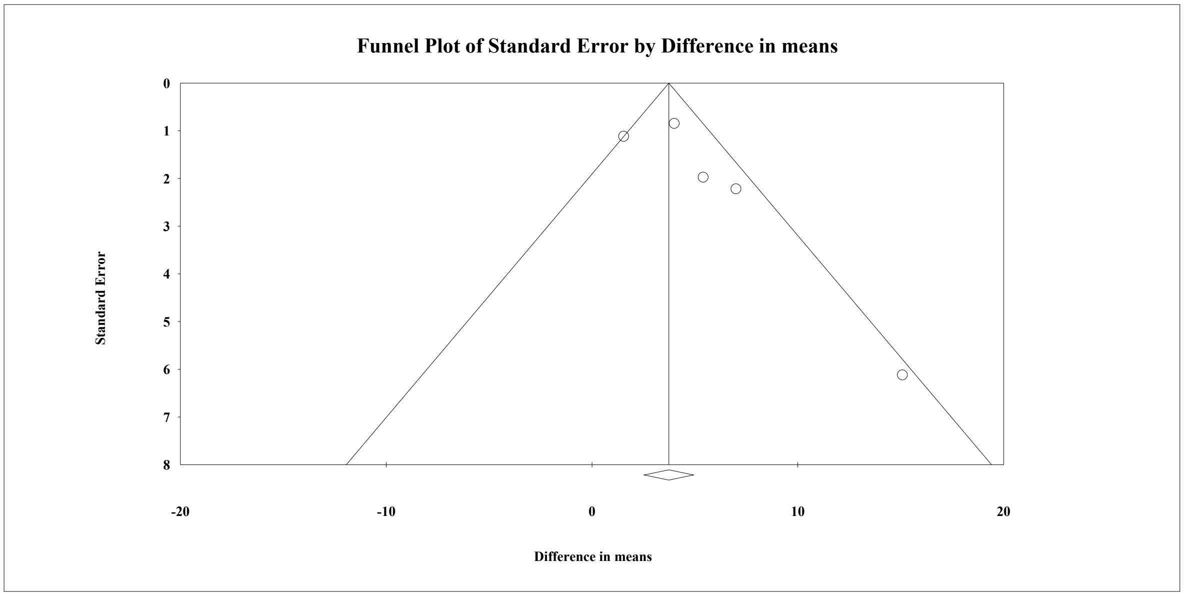

2.8. Supplementary Figure S8. Funnel plot for publication bias among studies reporting LDL-C after 3-month administration of LET. Visual inspection of funnel plot reveals asymmetry and presence of publication bias. LDL-C low-density lipoprotein cholesterol, LET letrozole.

Group by Subgroup within study	Study name	Difference in means	Lower limit	Upper limit	p-Value	Relative weight
ANAstrozole	Tian W et al, 2017	-3.480	-14.723	7.763	0.544	100.00
ANAstrozole		-3.480	-14.723	7.763	0.544	
EXEmestane	Gatti-Mays ME et al, 2016	-3.600	-17.175	9.975	0.603	16.22
EXEmestane	Markopoulos C et al, 2008	-1.000	-17.086	15.086	0.903	14.41
EXEmestane	Tian W et al, 2017	4.910	-11.734	21.554	0.563	14.03
EXEmestane	Markopoulos C et al, 2009	16.000	2.961	29.039	0.016	16.62
EXEmestane	Francini G et al, 2006	21.900	13.163	30.637	0.000	19.82
EXEmestane	Montagnani A et al, 2008	24.900	14.908	34.892	0.000	18.91
EXEmestane		11.667	1.851	21.484	0.020	
LETrozole	Zidan J et al, 2010	1.000	-0.968	2.968	0.319	97.11
LETrozole	Tian W et al, 2017	5.800	-5.608	17.208	0.319	2.89
LETrozole		1.139	-0.801	3.078	0.250	
TMX, tamoxifen	Markopoulos C et al, 2008	-23.000	-37.031	-8.969	0.001	30.44
TMX, tamoxifen	Montagnani A et al, 2008	-2.700	-15.335	9.935	0.675	32.36
TMX, tamoxifen	Francini G et al, 2006	1.160	-7.930	10.250	0.802	37.21
TMX, tamoxifen		-7.442	-21.262	6.378	0.291	

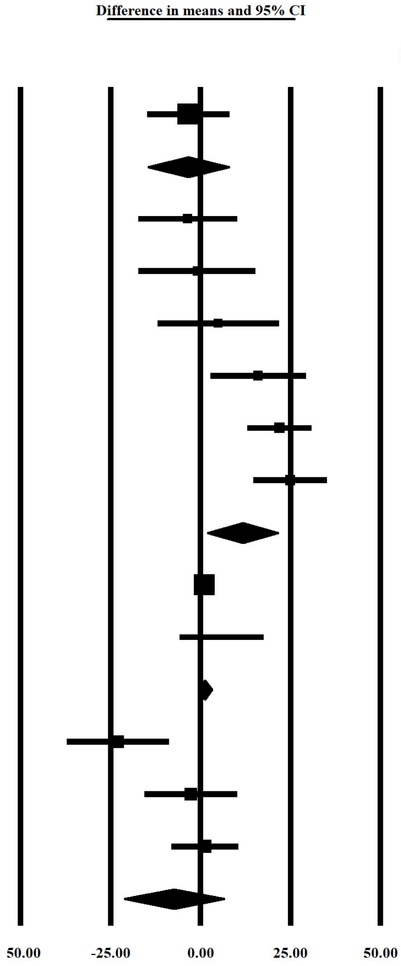

2.9. Supplementary Figure S9. Forest plot of LDL-C after 12-month administration of AIs and TMX. Pooled differences in the means were calculated between the baseline and endpoint treatment times for each study. Studies here are stratified by AIs (ANA, EXE, LET) and TMX measured by mg/dl (P<0.05). Weight contribution of studies is represented by the size of each box. The vertical lines represent the points of summary for random effect model, diamonds represent overall differences in the means of LDL-C for each strata. AI aromatase inhibitor, ANA anastrozole [44], CI confidence interval, EXE exemestane [31-34,42,44], LDL-C low-density lipoprotein, LET letrozole [36,44], TMX tamoxifen [31-33].

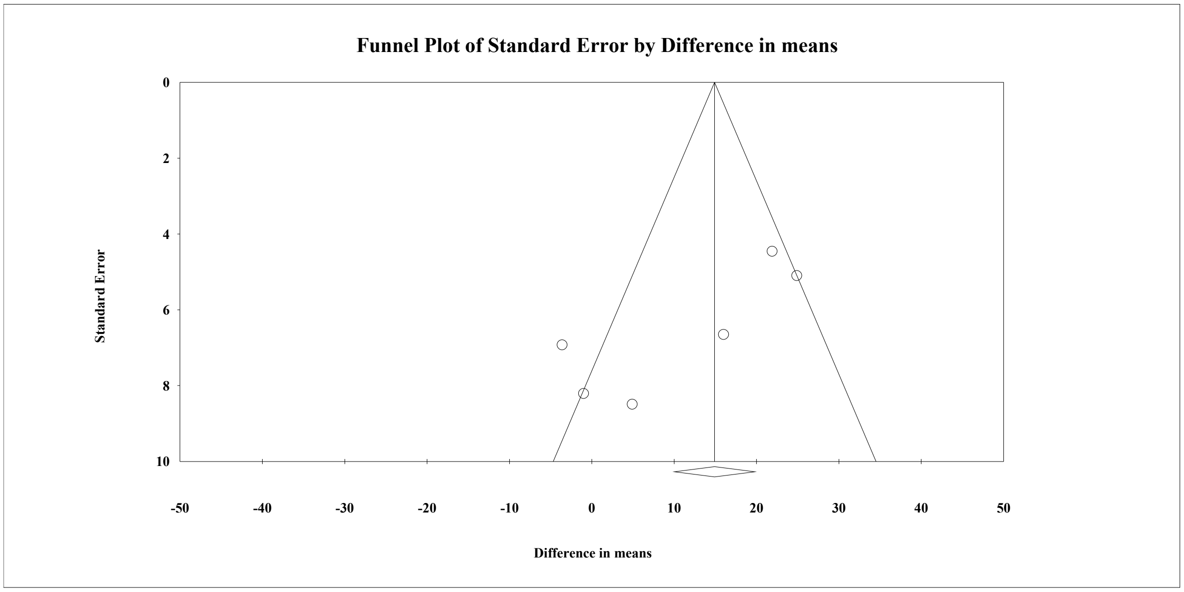

2.10. Supplementary Figure S10. Funnel plot for publication bias among studies reporting LDL-C after 12-month administration of EXE. Visual inspection of funnel plot reveals symmetry and absence of publication bias. EXE exemestane, LDL-C low-density lipoprotein cholesterol.

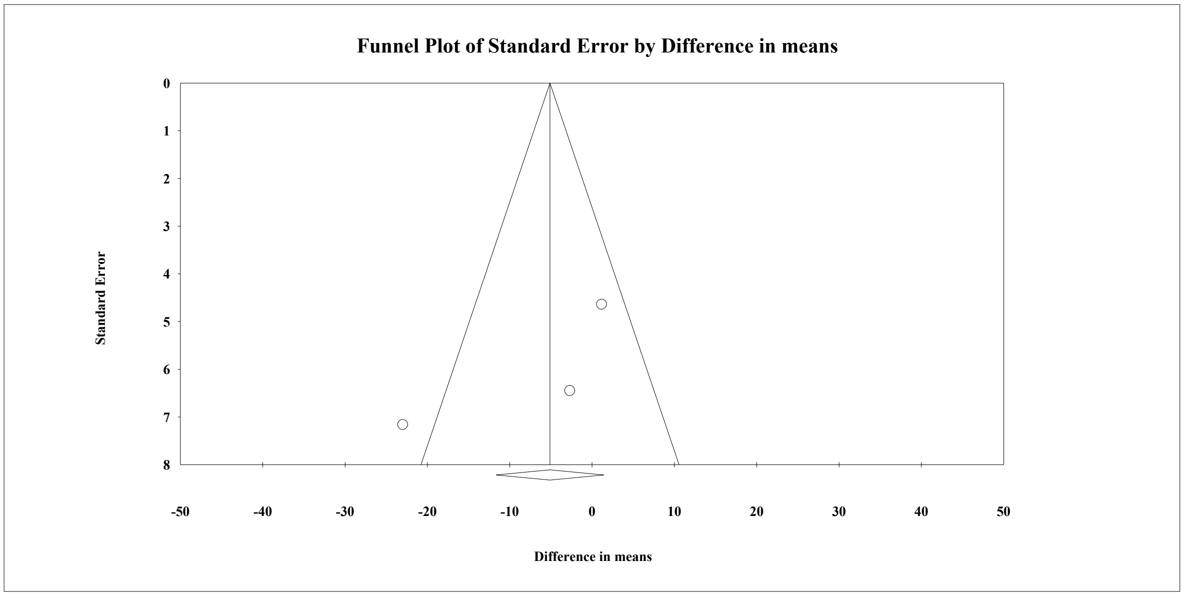

2.11. Supplementary Figure S11. Funnel plot for publication bias among studies reporting LDL-C after 12-month administration of TMX. Visual inspection of funnel plot reveals symmetry and absence of publication bias. LDL-C low-density lipoprotein cholesterol, TMX tamoxifen.

Group by Subgroup within study	Study name	Difference in means	Lower limit	Upper limit	p-Value	Relative weight
ANAstrozole	Tian W et al, 2017	3.480	-1.984	8.944	0.212	24.10
ANAstrozole	Sawada S et al, 2009	6.000	2.921	9.079	0.000	75.90
ANAstrozole		5.393	2.710	8.075	0.000	
EXEmestane	Gatti-Mays ME et al, 2016	-8.000	-14.666	-1.334	0.019	16.05
EXEmestane	Bell LN et al, 2012	-8.000	-12.644	-3.356	0.001	33.07
EXEmestane	Santa-Maria CA et al, 2016	-7.900	-12.500	-3.300	0.001	33.71
EXEmestane	Tian W et al, 2017	-2.700	-9.145	3.745	0.412	17.17
EXEmestane		-7.056	-9.727	-4.386	0.000	
LETrozole	López AM et al, 2015	-1.378	-4.469	1.713	0.382	16.09
LETrozole	Santa-Maria CA et al, 2016	-1.200	-2.534	0.134	0.078	19.07
LETrozole	Bell LN et al, 2012	-1.000	-2.417	0.417	0.167	18.97
LETrozole	Al-Biati Ha et al, 2017	0.470	-2.556	3.496	0.761	16.22
LETrozole	Tian W et al, 2017	3.090	-3.300	9.480	0.343	9.88
LETrozole	Zidan J et al, 2010	4.000	3.462	4.538	0.000	19.77
LETrozole		0.532	-2.298	3.362	0.712	
TMX, tamoxifen	Sawada S et al, 2009	2.000	-2.252	6.252	0.357	43.50
TMX, tamoxifen	Al-Biati Ha et al, 2017	3.080	-0.650	6.810	0.106	56.50
TMX, tamoxifen		2.610	-0.194	5.414	0.068	

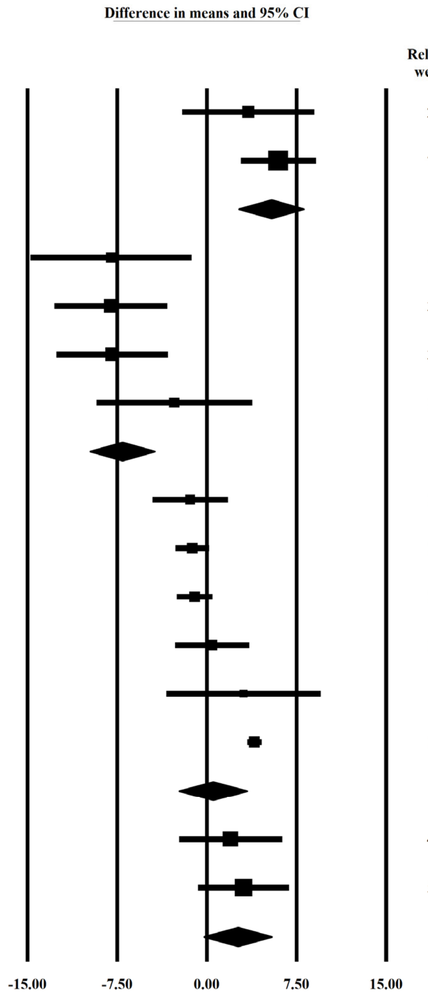

2.12. Supplementary Figure S12. Forest plot of HDL-C after 3-month administration of AIs and TMX. Pooled differences in the means were calculated between the baseline and endpoint treatment times for each study. Studies here are stratified by AIs (ANA, EXE, LET) and TMX measured by mg/dl (P<0.05). Weight contribution of studies is represented by the size of each box. The vertical lines represent the points of summary for random effect model, diamonds represent overall differences in the means of HDL-C for each strata. AI aromatase inhibitor, ANA anastrozole [35,44], CI confidence interval, EXE exemestane [38,40,42,44], HDL-C high-density lipoprotein, LET letrozole [36,38,40,41,43,44], TMX tamoxifen [35,43].

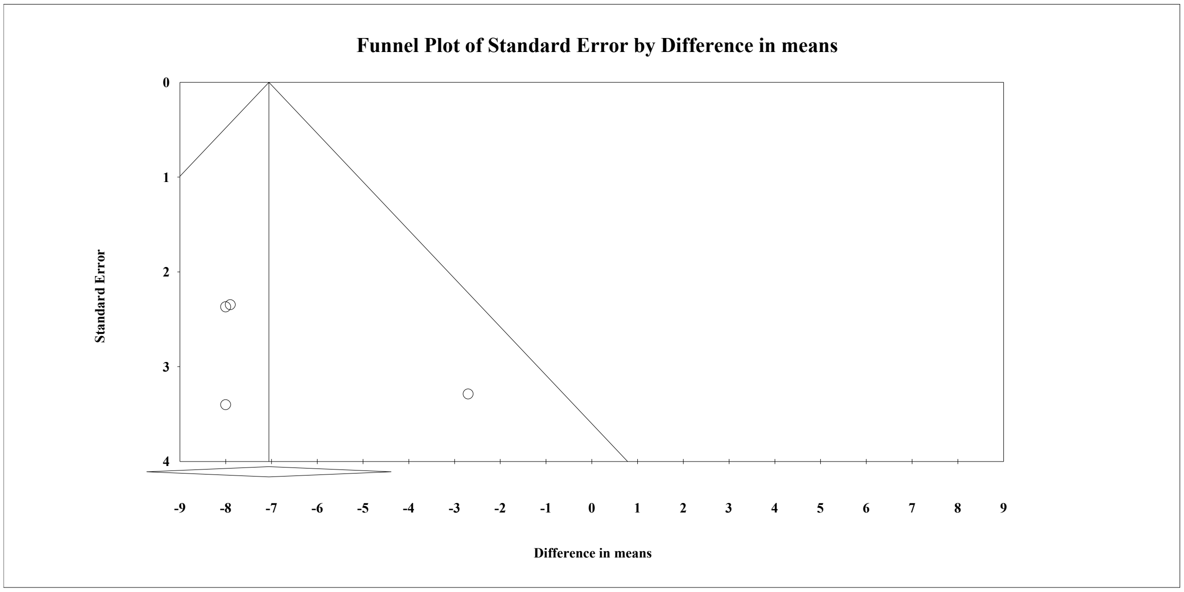

2.13. Supplementary Figure S13. Funnel plot for publication bias among studies reporting HDL-C after 3-month administration of EXE. Visual inspection of funnel plot reveals asymmetry and presence of publication bias. EXE exemestane, HDL-C high-density lipoprotein cholesterol.

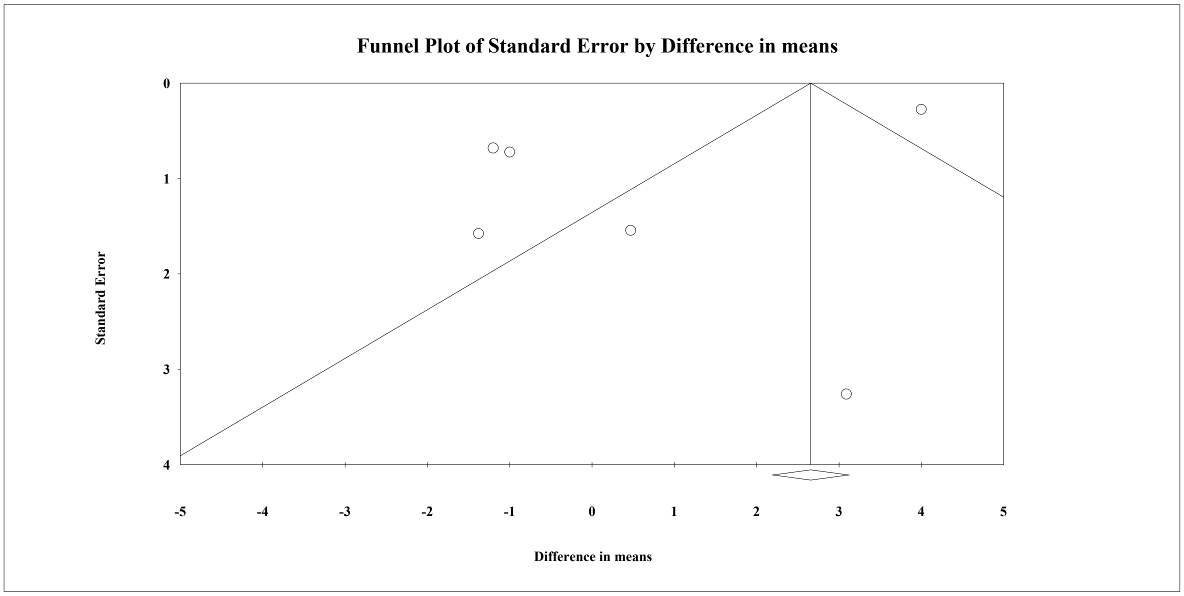

2.14. Supplementary Figure S14. Funnel plot for publication bias among studies reporting HDL-C after 3-month administration of LET. Visual inspection of funnel plot reveals asymmetry and presence of publication bias. HDL-C high-density lipoprotein cholesterol, LET letrozole.

Group by Subgroup within study	Study name	Difference in means	Lower limit	Upper limit	p-Value	Relative weight
ANAstrozole	Tian W et al, 2017	2.320	-3.203	7.843	0.410	100.00
ANAstrozole		2.320	-3.203	7.843	0.410	
EXEmestane	Montagnani A et al, 2008	-12.900	-15.198	-10.602	0.000	23.88
EXEmestane	Gatti-Mays ME et al, 2016	-8.600	-15.481	-1.719	0.014	15.33
EXEmestane	Francini G et al, 2006	-6.870	-10.688	-3.052	0.000	21.26
EXEmestane	Markopoulos C et al, 2008	-4.000	-9.407	1.407	0.147	18.13
EXEmestane	Markopoulos C et al, 2009	-4.000	-7.745	-0.255	0.036	21.40
EXEmestane		-7.441	-11.686	-3.195	0.001	
LETrozole	Zidan J et al, 2010	2.000	1.245	2.755	0.000	98.70
LETrozole	Tian W et al, 2017	5.020	-1.561	11.601	0.135	1.30
LETrozole		2.039	1.289	2.790	0.000	
TMX, tamoxifen	Montagnani A et al, 2008	-1.000	-6.288	4.288	0.711	34.22
TMX, tamoxifen	Francini G et al, 2006	-0.640	-4.454	3.174	0.742	65.78
TMX, tamoxifen		-0.763	-3.856	2.330	0.629	

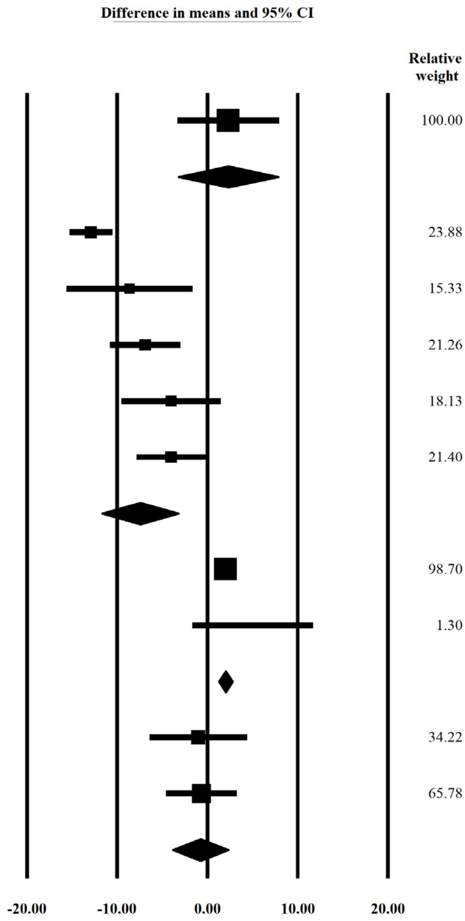

2.8.15. Supplementary Figure S15. Forest plot of HDL-C after 12-month administration of AIs and TMX. Pooled differences in the means were calculated between the baseline and endpoint treatment times for each study. Studies here are stratified by AIs (ANA, EXE, LET) and TMX measured by mg/dl (P<0.05). Weight contribution of studies is represented by the size of each box. The vertical lines represent the points of summary for random effect model, diamonds represent overall differences in the means of HDL-C for each strata. AI aromatase inhibitor, ANA anastrozole [44], CI confidence interval, EXE exemesthane [31-34,42], HDL-C high-density lipoprotein, LET letrozole [36,44], TMX, tamoxifen [31,32].

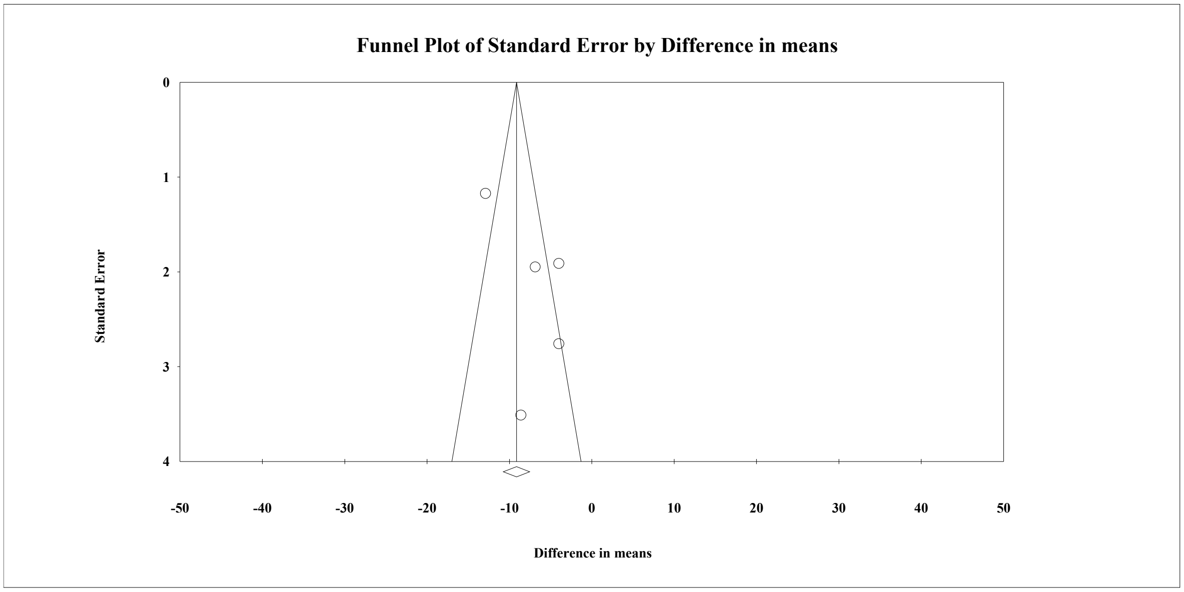

2.8.16. Supplementary Figure S16. Funnel plot for publication bias among studies reporting HDL-C after 12-month administration of EXE. Visual inspection of funnel plot reveals symmetry and absence of publication bias. EXE exemestane, HDL-C high-density lipoprotein cholesterol.

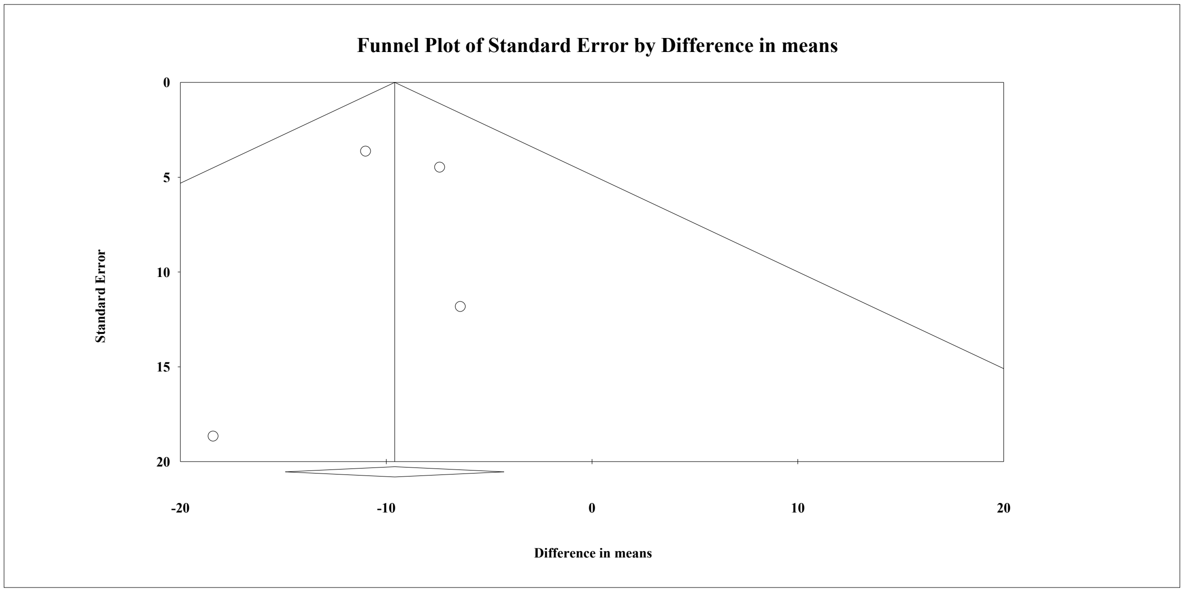

2.8.17. Supplementary Figure S17. Funnel plot for publication bias among studies reporting TG after 3-month administration of EXE. Visual inspection of funnel plot reveals symmetry and absence of publication bias. EXE exemestane, TG triglycerides.

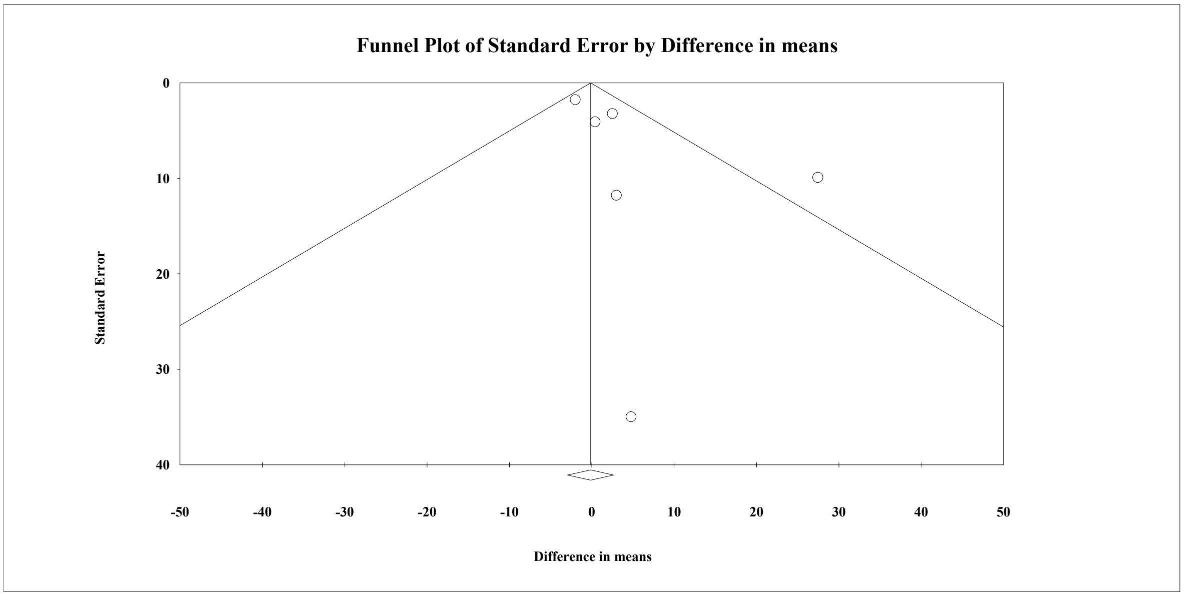

2.8.18. Supplementary Figure S18. Funnel plot for publication bias among studies reporting TG after 3-month administration of LET. Visual inspection of funnel plot reveals asymmetry and presence of publication bias. LET letrozole, TG triglycerides.

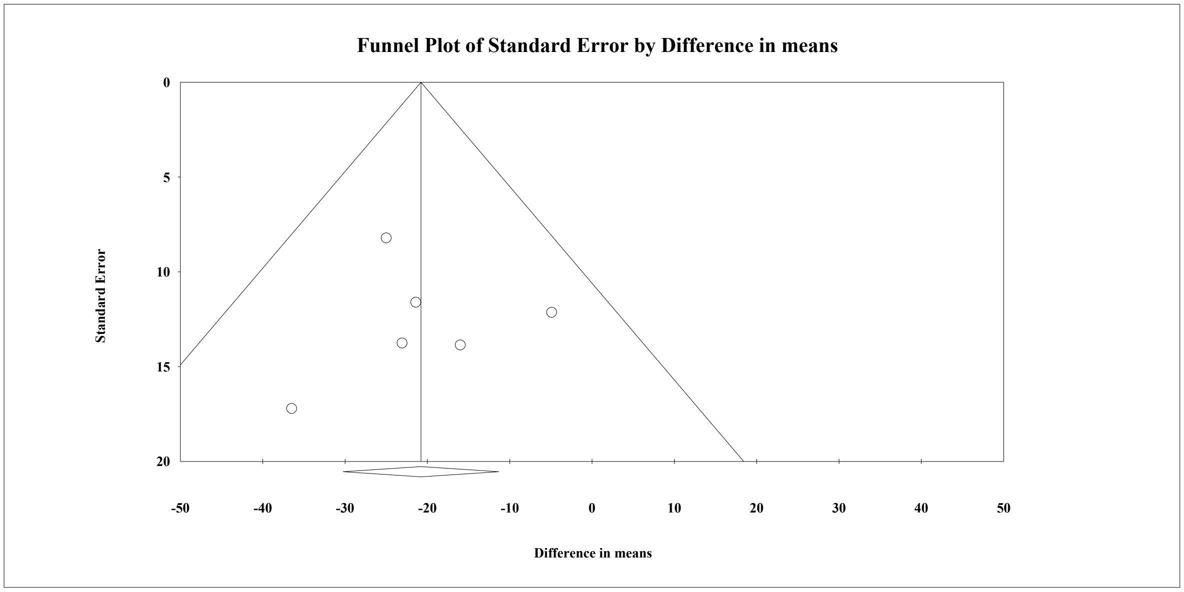

2.8.19. Supplementary Figure S19. Funnel plot for publication bias among studies reporting TG after 12-month administration of EXE. Visual inspection of funnel plot reveals symmetry and absence of publication bias. EXE exemestane, TG triglycerides.

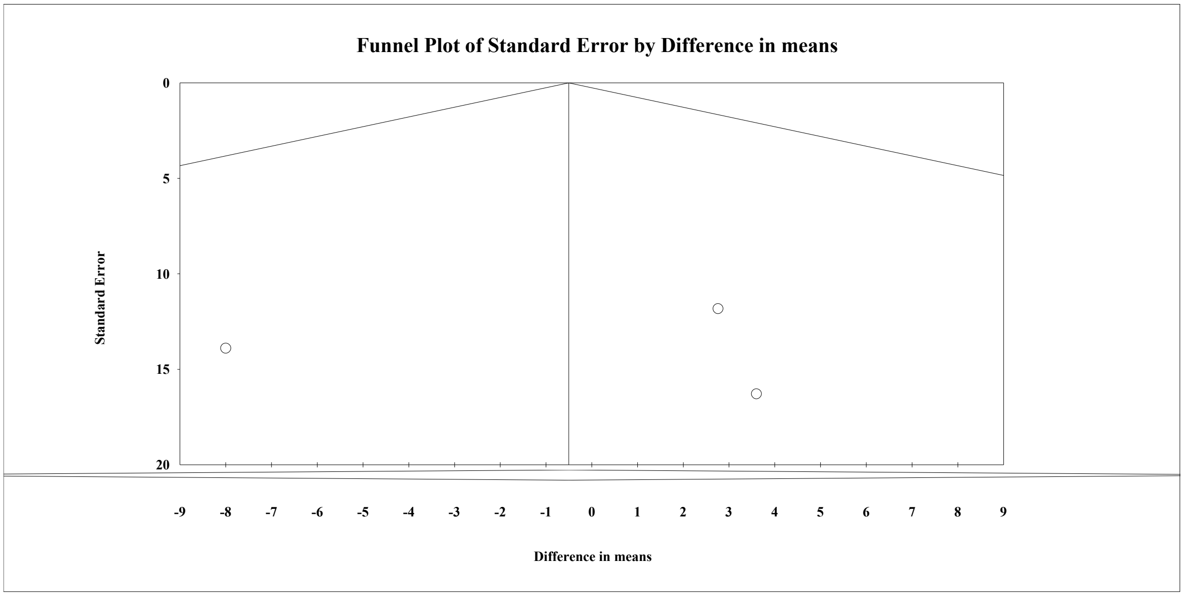

2.8.20. Supplementary Figure S20. Funnel plot for publication bias among studies reporting TG after 12-month administration of TMX. Visual inspection of funnel plot reveals symmetry and absence of publication bias. TG triglycerides, TMX tamoxifen.

3. References

1. Lønning, PE. et al. Effects of exemestane administered for 2 years versus placebo on bone mineral density, bone biomarkers, and plasma lipids in patients with surgically resected early breast cancer. J. Clin. Oncol. 23, 5126-5137 (2005).
2. Francini, G. et al. Exemestane after tamoxifen as adjuvant hormonal therapy in postmenopausal women with breast cancer: effects on body composition and lipids. Br. J. Cancer. 95, 153-158 (2006).
3. Montagnani, A. et al. The effects on lipid serum levels of a 2-year adjuvant treatment with exemestane after tamoxifen in postmenopausal women with early breast cancer. Eur. J. Intern. Med. 19, 592-597 (2008).
4. Markopoulos, C. et al. Lipid changes in breast cancer patients on exemestane treatment: final results of the TEAM Greek substudy. Ann. Oncol. 20, 49-55 (2009).
5. Markopoulos, C. et al. Extended adjuvant hormonal therapy with exemestane has no detrimental effect on the lipid profile of postmenopausal breast cancer patients: final results of the ATENA lipid substudy. Breast Cancer Res. 11, R35 (2009).
6. Sawada, S. et al. Effect of anastrozole and tamoxifen on lipid metabolism in Japanese postmenopausal women with early breast cancer. Acta. Oncol. 44, 134-141 (2005).
7. Zidan, J., Chetver, L., Hussein, O., Zucker, M. Effect of letrozole on plasma lipids, triglycerides, and estradiol in postmenopausal women with metastatic breast cancer. Oncologist. 15, 1159-1163 (2010).
8. Anan, K. et al. Effects of toremifene and anastrozole on serum lipids and bone metabolism in postmenopausal females with estrogen receptor-positive breast cancer: the results of a 2-year multicenter open randomized study. Breast Cancer Res. Treat. 128, 775-781 (2011).
9. Bell LN. et al. Comparison of changes in the lipid profile of postmenopausal women with early stage breast cancer treated with exemestane or letrozole. J. Clin. Pharmacol. 52, 1852-1860 (2012).

10. Iwata, H. et al. A randomized, double-blind, controlled study of exemestane versus anastrozole for the first-line treatment of postmenopausal Japanese women with hormone-receptor-positive advanced breast cancer. Breast Cancer Res. Treat. 139, 441-451 (2013).
11. Santa-Maria, CA. et al. Association of variants in candidate genes with lipid profiles in women with early breast cancer on adjuvant aromatase inhibitor therapy. Clin Cancer Res. 22, 1395-1402 (2016).
12. López, AM. et al. Double-blind, randomized trial of alternative letrozole dosing regimens in postmenopausal women with increased breast cancer risk. Cancer Prev. Res. 9, 142-148 (2016).
13. Gatti-Mays, ME. et al. Exemestane use in postmenopausal women at high risk for invasive breast cancer: evaluating biomarkers of efficacy and safety. Cancer Prev. Res. 9, 225-233 (2016).
14. Al-Biati, HA., Sahib, AS., Mahmood, AN. Effects of tamoxifen or letrozole on lipid profile, vitamin D and estradiol serum levels in obese postmenopausal woman with breast cancer. Int. J. Pharm. Sci. 9, 142-144 (2017).
15. Tian, W., Wu, M., Deng, Y. Comparison of changes in the lipid profiles of Eastern Chinese postmenopausal women with early-stage breast cancer treated with different aromatase inhibitors: A retrospective study. Clin. Pharmacol. Drug Dev. 7, 837-843 (2018).
